# Near Infrared Biomimetic Hybrid Magnetic Nanocarrier for MRI-Guided Thermal Therapy

**DOI:** 10.1021/acsami.4c03434

**Published:** 2024-07-08

**Authors:** João Victor Ribeiro Rocha, Rafael Freire Krause, Carlos Eduardo Ribeiro, Nathália Corrêa de Almeida Oliveira, Lucas Ribeiro de Sousa, Juracy Leandro Santos, Samuel de Melo Castro, Marize Campos Valadares, Mauro Cunha Xavier Pinto, Marcilia Viana Pavam, Eliana Martins Lima, Sebastião Antônio Mendanha, Andris Figueiroa Bakuzis

**Affiliations:** † Institute of Physics, Federal University of Goiás, Goianiâ, Goiás 74690-900, Brazil; ‡ FarmaTec − Laboratory of Pharmaceutical Technology, Federal University of Goiás, Goianiâ, Goiás 74690-631, Brazil; § ToxIn − Laboratory of Education and Research in In Vitro Toxicology, Federal University of Goiás, Goianiâ, Goiás 74690-631, Brazil; ∥ Department of Pharmacology, Institute of Biological Sciences, Federal University of Goiás, Goianiâ, Goiás 74690-900, Brazil; ⊥ CNanoMed − Nanomedicine Integrated Research Center, Federal University of Goiás, Goianiâ, Goiás 74690-631, Brazil

**Keywords:** thermal nanomedicine, cell membrane nanoparticles, SPION, cancer, glioblastoma.

## Abstract

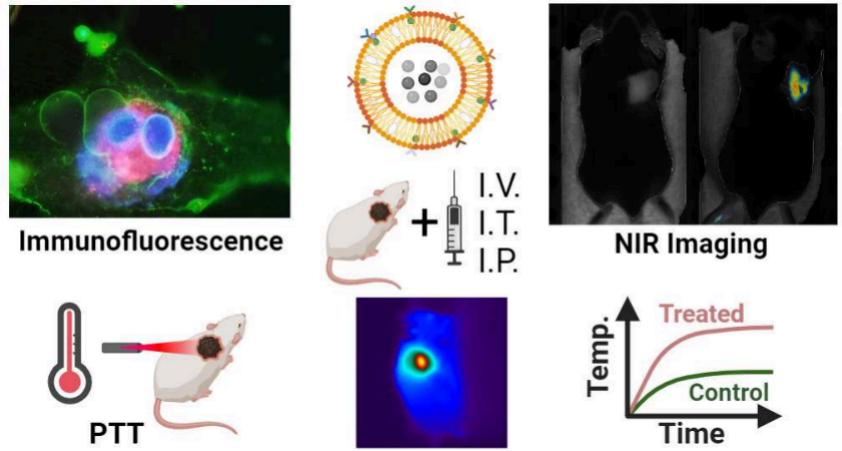

Cell-membrane hybrid nanoparticles (NPs) are designed to improve drug delivery, thermal therapy, and immunotherapy for several diseases. Here, we report the development of distinct biomimetic magnetic nanocarriers containing magnetic nanoparticles encapsulated in vesicles and IR780 near-infrared dyes incorporated in the membranes. Distinct cell membranes are investigated, red blood cell (RBC), melanoma (B16F10), and glioblastoma (GL261). Hybrid nanocarriers containing synthetic lipids and a cell membrane are designed. The biomedical applications of several systems are compared. The inorganic nanoparticle consisted of Mn-ferrite nanoparticles with a core diameter of 15 ± 4 nm. TEM images show many multicore nanostructures (∼40 nm), which correlate with the hydrodynamic size. Ultrahigh transverse relaxivity values are reported for the magnetic NPs, 746 mM^–1^s^–1^, decreasing respectively to 445 mM^–1^s^–1^ and 278 mM^–1^s^–1^ for the B16F10 and GL261 hybrid vesicles. The ratio of relaxivities *r*_2_/*r*_1_ decreased with the higher encapsulation of NPs and increased for the biomimetic liposomes. Therapeutic temperatures are achieved by both, magnetic nanoparticle hyperthermia and photothermal therapy. Photothermal conversion efficiency ∼25–30% are reported. Cell culture revealed lower wrapping times for the biomimetic vesicles. *In vivo* experiments with distinct routes of nanoparticle administration were investigated. Intratumoral injection proved the nanoparticle-mediated PTT efficiency. MRI and near-infrared images showed that the nanoparticles accumulate in the tumor after intravenous or intraperitoneal administration. Both routes benefit from MRI-guided PTT and demonstrate the multimodal theranostic applications for cancer therapy.

## I Introduction

Biomimetic nanocarriers are expected to have a great impact in the clinic, since coating the surface of nanoparticles (NPs) with cell membranes can enhance blood circulation time, improve cancer targeting, promote immune stimulations, among other things.^1^ On the other hand, choosing wisely the NP can allow theranostic applications since it might promote diagnosis and image-guided therapy. An interesting choice for core material is iron oxide-based nanoparticles because of several clinical applications, such as anemia treatment, MRI contrast agents, magnetic tracers for magnetic particle imaging (MPI) and alternating current biosusceptometry (ACB), photothermal therapy (PTT), and magnetic nanoparticle hyperthermia (MNH).^2−7^

Curiously, the cell-based research with magnetic nanomaterials are still rare.^1^ Although, it is clear that the association of magnetic nanoparticles with the biomimetic coating allows for improved theragnostic applications, as shown by several groups.^8−16^ For instance, Rao et al. demonstrated that surface coating magnetite large NPs (80 nm) with RBC membrane is superior than pegylated NPs, due to longer circulation time and absence of humoral immune response, even after multiple injections.^8,9^

In addition, MRI-guided thermal therapy with magnetic NPs is proposed by Lai et al. for an *in vitro* prostate cancer MNH study.^17^ The transverse relaxivity (*r*_2_) of the membrane-coated magnetite NP (653 mM^–1^ s^–1^) was found to be lower than that of the citrate-coated NP (786 mM^–1^ s^–1^). Ren et al. describe *in vivo* breast cancer PTT study.^18^ The relaxivity was found to be 1 order of magnitude lower, but a similar phenomenon is reported (*r*_2_ decreased from 82 mM^–1^ s^–1^ to 75 mM^–1^ s^–1^ for the biomimetic NP).

Yu et al. showed evidence of PTT-induced immunogenic cell death (ICD) and macrophage polarization in a breast cancer study.^11^ Indeed, it was recently proved that biodegradation of iron oxide NPs results in lower tumor growth rates and less metastasis focus due to this polarization.^19^ Since NPs uptake depend on macrophage polarization,^20^ one might expect important diagnosis applications, using MPI, ACB or MRI.

MRI-guided triple modal therapy has been explored recently by Mo et al.^21^ Albumin-coated gold nanorods encapsulated with Fe^3+^ coupled to doxorubicin (DOX) was investigated. Gold nanorods were used due to the excellent PTT response, DOX for chemotherapy, and intracellular Fe^3+^ release for chemodynamic therapy (CDT) mediated by Fenton reactions and MRI-guidance. Multimodal and MRI-guided therapeutic strategies are hot topics^22,23^ and could be enhanced with cell-membrane technology. Other approaches using distinct materials have been explored, but one should keep in mind that several are not approved for clinical use in contrast to iron oxide NPs, which could also benefit from CDT due to particle biodegradation.

Near infrared (NIR) imaging capabilities with magnetic nanomaterials are even more rare. Bose et al. report the use of ICG (FDA approved NIR agent) in 4T1-coated magnetite NPs^24^ and show *in vivo* MRI images. Wang et al. use IR780 in RBC-coated magnetite NPs.^25^ IR780, although not approved in the clinic, has superior optical properties in comparison to ICG, being valuable for imaging, photodynamic therapy (PDT), and PTT.^26^

Our group has explored NIR RBC-coated magnetic NPs for pharmacokinetics (PK), biodistribution and PTT cancer studies, mainly in sarcoma (S180) and breast carcinoma (Ehrlich).^12,16^ Mn-doped iron oxide NPs were used due to excellent low field MNH and ACB properties.^5,27−32^ In the PK study we described *in vitro* MNH results, but at the time, low magnetic hyperthermia efficiency at clinically relevant conditions was reported.^12^ On the other hand, we demonstrate PTT-induced immunogenic cell death (ICD), resulting in an extremely high survival rate in the S180 murine tumor model.^16^

The literature shows that RBC membranes are been the most popular choice in order to grafting a stealth coating layer onto the surface of NPs,^1^ while only few cancer membranes had been used to coat magnetic NPs.^14,15,24,33^ Indeed, there is no report for coating magnetic NPs with neither, melanoma or glioblastoma (GBL) membranes, although melanoma membranes have already been used in the coating of other type of particles such as CuS or PLGA NPs.^34,35^

GBL is the most aggressive brain cancer with a very poor prognosis. The median survival rate after diagnosis is around 15 months. So far, the standard of care for patients is surgery, radiotherapy, corticosteroids and chemotherapy (mainly using Temozolomide), which might have immunosuppressive effects.^36^ Photothermal-induced strategies are being developed to modulate the blood–brain–tumor barrier permeability and increase the possibility to other drugs.^37^ On the other hand, MNH is approved for treating brain tumors together with radiotherapy after NPs intratumoral administration.^38^ Efficient low field nanoheaters might allow a single thermal therapy modality.^7^ Due to the lack of therapy efficacy with traditional approaches, there is hope that immunotherapy might play a role in this type of cancer, resulting in better outcomes.^36^

Magnetic nanoparticles and thermal therapy are promising strategies for immunotherapy.^19,39^ NPs can biodegrade releasing metallic ions that are important for many immunological processes,^40^ while heat can trigger many innate and adaptive responses.^39^ Therefore, the encapsulation of this kind of NPs in smart nanocarriers emerges as an excellent aproach toward GBL or melanoma.

In this article, we explore the biomimetic magnetic coating strategy. We encapsulate Mn-ferrite nanoparticles into cell-membrane-hybrid liposomes composed of different membrane fragments (RBC, B16F10, or GL261) enriched with extrinsic lipids and incorporate IR780 dyes into their membranes. Several techniques are used for NP characterization, which allows detailed connection between structure-related MRI, PTT, and MNH properties. *In vitro* studies demonstrate the lower wrapping time of the hybrid vesicles, while *in vivo* experiments are added as a proof of concept for an image-guided therapeutic procedure. The goal of the study is to develop novel theranostic biomimetic NPs for image-guided thermal therapy at clinically relevant conditions.

## II Results and Discussions

### II.A Hybrid Biomimetic Design for Theranostics

**Figure 1:**
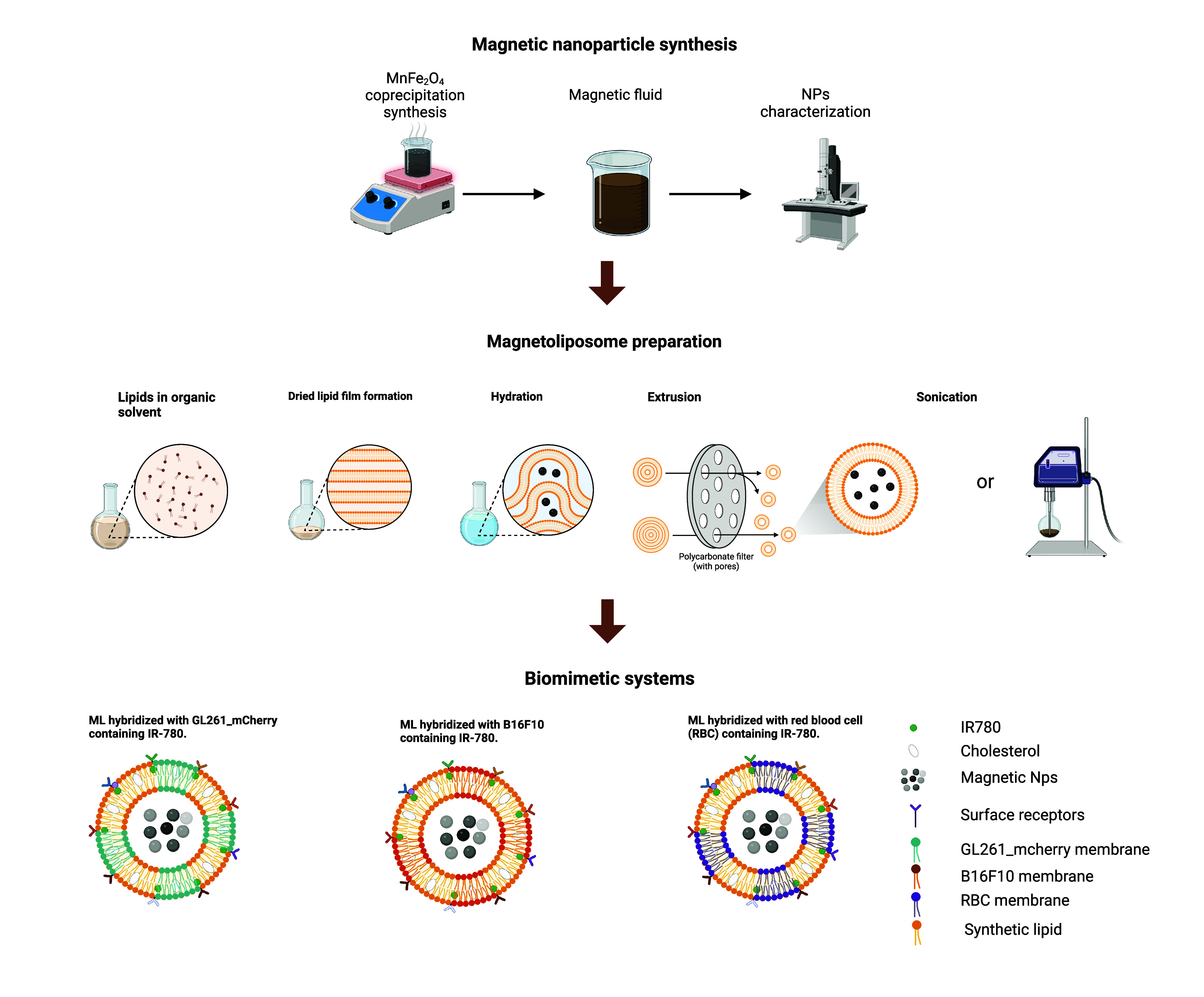
Representative scheme of the steps for production of the biomimetic magnetofluorescent liposomes.

Biomimetic magnetoliposomes (ML) derived from cancer cells of GL261 and B16F10 were developed and characterized in this work. For comparison, an additional ML hybridized with red blood cells (RBC) membranes was also included in our studies, while the general procedure to obtain biomimetic vesicles can be found in reference.^41^ The final MLs particle concentrations were tuned to obtain a higher (or lower) NPs encapsulation inside the vesicles using a Mn-ferrite nanoparticle colloidal suspension of different magnetic fractions. In addition, MLs were subjected to sonication or extrusion in order to modulate vesicle lamellarity and polydispersity. This procedure also modulates NPs encapsulation. Mn-ferrite nanoparticles were synthesized by the coprecipitation method and were surface-coated with citrate for stability at physiological conditions, as previously discussed elsewhere.^31^Figure 1 shows a scheme of the method of the synthesis of magnetic NPs and the preparation of hybrid magnetoliposomes.

**Figure 2:**
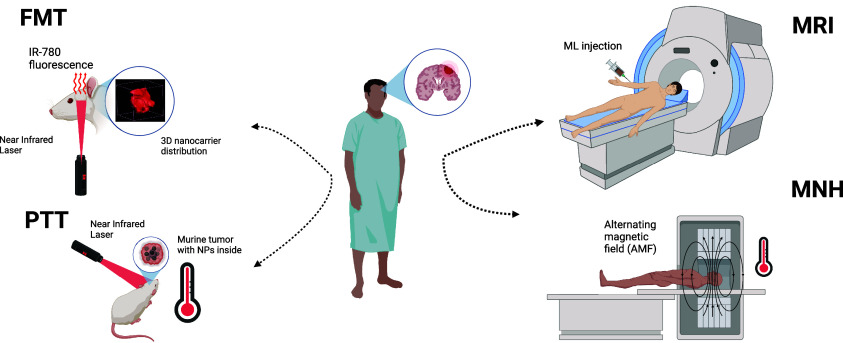
Cancer theranostic applications of hybrid magnetofluorescent liposomes. FMT - Fluorescence molecular tomography, MRI - Magnetic resonance imaging, PTT - Photothermal therapy, MNH - Magnetic nanoparticle hyperthermia.

All over the years, magnetoliposomes were design as nanocarriers and their functionalities explore mainly the controlled drug release over external magnetic stimulus.^42,43^ Additionally, one could also find their use as MRI contrast agent.^44^ However, even though biomimetic liposomes being recognized as the state of the art when it comes to multifunctional nanocarriers, with potential applicability on the prevention, treatment, and detection of different types of cancer, infectious and inflammatory diseases,^45−47^ to our knowledge, there are no reports of magnetoliposomes designed as multifunctional biomimetic vesicles based on melanoma or glioblastoma cell membranes. The potential theranostic applications of our hybrid biomimetic vesicles are listed in Figure 2.

### II.B NPs Encapsulation Depend on the Hydration Step

**Figure 3:**
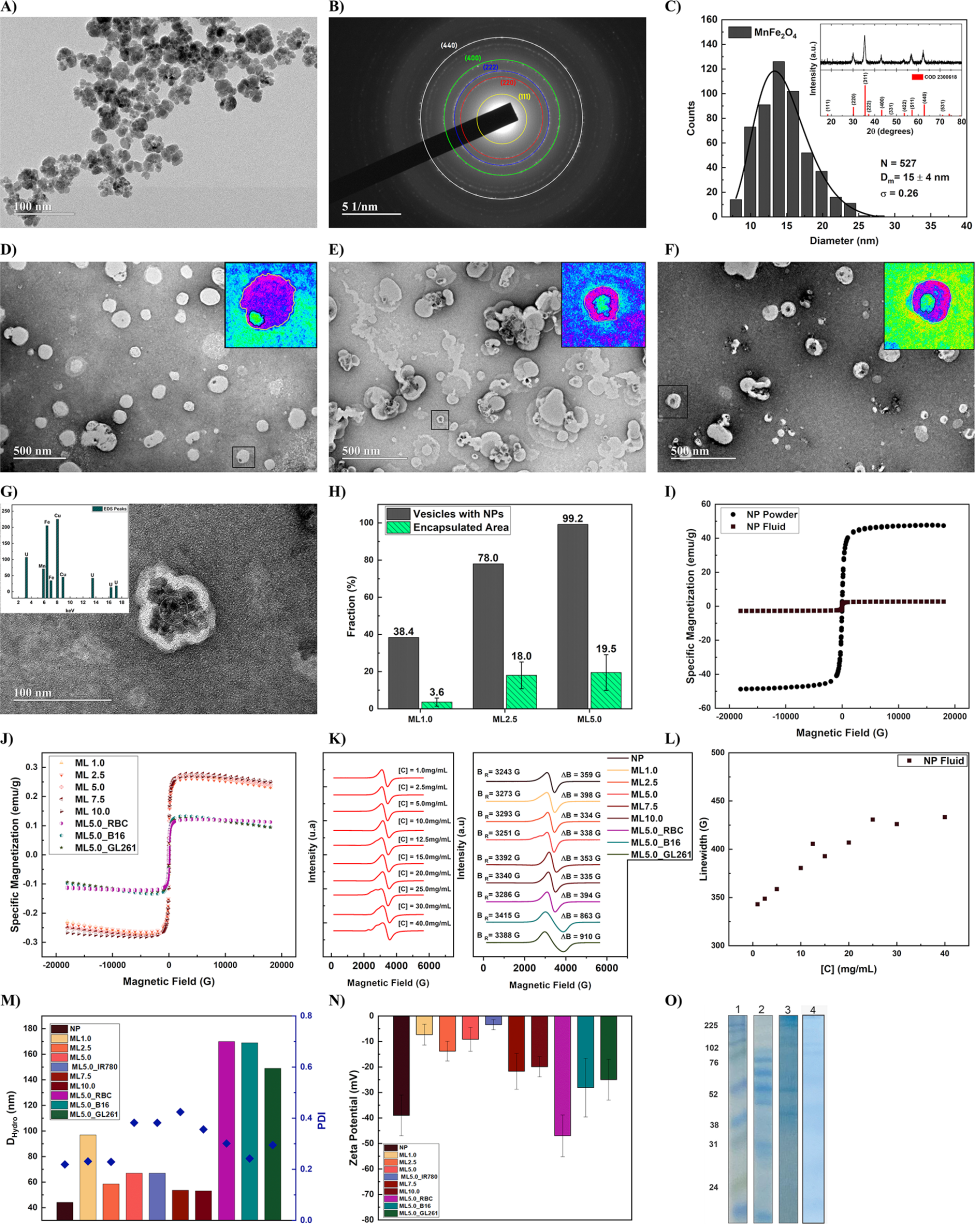
(A) Representative TEM images of the Mn-ferrite NPs. (B) SAED. (C) TEM Mn-ferrite size distribution. The inset shows the XRD of the NPs. (D) TEM image of ML1.0. (E) TEM image of ML2.5. (F) TEM image of ML5.0. The insets of Figures D-F show isolated MLs evaluated using ImageJ for area determination. (G) High resolution TEM image of isolated ML showing the encapsulation of NPs. The inset shows the EDS data of this ML. (H) Fraction of vesicles containing NPs and area occupied by NPs encapsulated in the vesicles for samples ML1.0, ML2.5, and ML5.0. (I) Specific magnetization curve of the Mn-ferrite NPs and of their colloidal suspension. (J) Specific magnetization curves of the magneto vesicles. (K) ESR spectra of Mn-ferrite colloidal suspension at different concentrations (left) and MLs samples at fixed NP concentration (right). (L) ESR line width concentration dependence of the Mn-ferrite magnetic fluid. (M) MLs mean size and polydispersity obtained by DLS. (N) MLs zeta potential profile. (O) SDS-PAGE of representative hybrid liposomes: 1 molecular weight markers; 2 melanoma (B16F10); 3 glioblastoma (GL261) and 4 red blood cells (RBC) cell-derived liposomes protein profile indicating the cell-membrane fragments retention by each hybrid vesicle.

Figure 3(A) shows a typical TEM image of the citrate-coated NPs. One can observe several multicore nanostructures (∼40 nm), with core nanoparticles of spherical shape (∼10 nm). Figure 3(B) shows the selected area electron diffraction (SAED) patterns, which confirms the crystalline phase of the NPs. It was observed the following planes of spinel structure, (111), (220), (222), (400), and (440). Similar planes are observed by XRD; see the inset of Figure 3(C). The crystallite size was estimated using Scherrer’s equation and found to be 14.4 nm. From the analysis of several TEM pictures one can extract the diameter profile. Figure 3(C) shows the TEM size histogram of the NPs, revealing a log-normal size distribution with a mean diameter of 15 ± 4 nm.

Figure 3(D), (E), and (F) shows representative TEM images of the magnetoliposomes (MLs) prepared by extrusion, considering distinct NP concentration during the hydration step, namely, 1.0 (ML1.0), 2.5 (ML2.5), and 5.0 (ML5.0) mg/mL, respectively. One observes aggregated NPs inside the vesicles in a concentration dependent manner. Figure 3(G) shows a TEM image at higher resolution, while the inset shows the EDS result. Original EDS data can be found in the SI, Figure S1. Since the samples were deposited on carbon films of a TEM copper grid and colored by 0.5% aqueous uranyl acetate, it is easy to understand why we observe uranyl. More importantly, the data also show Fe and Mn, which confirms the encapsulation of Mn-doped iron oxide NPs. The images of Figure 3(D), (E), and (F) clearly reveal that for the lower concentration the number of vesicles without any NP is higher. An analysis of the TEM images reveals that the fraction of liposomes with NPs, shown in Figure 3(H), grows from 38 to almost 100% from 1.0 to 5.0 mg/mL. Figure S2 shows representative TEM images of the hybrid vesicles.

To better evaluate how the NPs are organized inside the vesicles, we performed an analysis using ImageJ. For this, we isolate separate vesicles containing NPs and with appropriate contours obtain the areas of the vesicle and the NPs encapsulated on them. The insets of Figure 3(D), (E), and (F) show a representative picture of this analysis, while on Figure 3(H) is shown the fraction of encapsulated NPs. The fraction increased from 3.6 to 19.5% increasing from 1.0 to 5.0 mg/mL. Assuming spherical NPs we can estimate the 3D particle volume fraction to be 1.27%, 6.55%, and 6.23% for the MLs prepared at 1.0, 2.5, and 5.0 mg/mL, respectively. The diameters of the vesicles used in the estimations appear in the SI in Figure S3.

### II.C Magnetic Characterization Suggests Distinct Particle Arrangements Inside Vesicles

Figure 3(I) shows the specific magnetization curve of the Mn-ferrite NPs, and the corresponding colloid. The curve shows a superparamagnetic-like behavior expected for this soft magnetic material at this size. From the specific saturation magnetization, one can calculate the magnetic particle volume fraction or the NP concentration using eq 3. For some characterization analysis, it might be relevant to maintain samples with similar concentrations. Figure 3(J) shows the specific magnetization curves of the MLs and hybrid magnetic vesicles. MLs are tuned to 5.0 mg/mL, while the HVs were set to 2.5 mg/mL. The magnetization curves show two contributions, one from the superparamagnetic NPs and another diamagnetic term due to the lipids and proteins.

Electron spin magnetic resonance (ESR) is used to investigate how the NPs organized and interacted inside the vesicles. Both, resonance field and resonance line width are known to depend on particle concentration.^31,48,49^ Difference between them correlates with distinct particle arrangements and degrees of interaction. Panels in Figure 3(K) show ESR spectra of Mn-ferrite colloid (left) and distinct samples (right), at fixed NP concentration, namely MLs and HVs (right). For the latter we included information about the resonance field and line width values. Indeed, Figure 3(L) shows the line width concentration dependence of the Mn-ferrite magnetic fluid. Clearly, an increase due to particle–particle interactions is observed. The behavior is well-known,^31,48^ and is related to dipolar broadening. Samples NP, ML2.5, ML5.0, ML7.5, and ML10 show line widths ∼340–360 G, ML1.0 and ML5.0_RBC ∼ 395 G, while ML5.0_B16F10 and ML5.0_GL261 900 G. From this data, it is easy to conclude that GL261 and B16F10 hybrid vesicles are very different from the NP or the synthetic liposomes, while ML1.0 and ML5.0_RBC show slight differences with the other samples. On the other hand, higher resonance field values might indicate a more random-like anisotropy axis configuration for the hybrid vesicles in comparison to the NP and MLs. This could arise from a higher NP encapsulation efficiency.

Figure 3(M) shows the hydrodynamic size of all the samples investigated, namely, the MLs prepared by sonication, and the hybrid magnetic vesicles. The result shows that the magnetic fluid has the lowest size, ∼40 nm. On the contrary, ML1.0, and the hybrid vesicles showed larger sizes. ML1.0 the size is ∼100 nm, while the HVs ∼150–170 nm. Note that this larger hydrodynamic size correlates with higher ESR line widths. On the other hand, samples ML2.5, ML5.0, ML7.5, and ML10, although have larger sizes in comparison to the NP, showed values lower than 100 nm. Since the samples were prepared through sonication, the DLS characterization suggests that those samples might have both NP configurations, dispersed in the liquid or encapsulated inside the liposome. Moreover, the increase in liposome mean size due its fusion with cell membrane fragments was previously reported by our group.^41^Figure 3(N) shows the zeta potential characterizations for all samples. As expected, encapsulating the NPs inside the vesicles results in a decrease on the surface charge. In addition, we also included a ML5.0_IR780 sample, where the near-infrared dye is incorporated after the ML preparation. One clearly observe a huge decrease of the zeta potential due to the positive charge of the ion of the heptamethine dye. Indeed, depending on the amount of IR780 incorporated into the membrane, a zeta potential can be tuned.

Finally, the protein profiles of biomimetic MLs are shown in Figure 3(O). Sodium dodecyl sulfate-polyacrylamide gel electrophoresis (SDS-PAGE) confirmed the successful of cell-membrane ML hybridization, demonstrating that the protein composition of biomimetic MLs was similar to those of cell membranes that they were derived and suggesting a good retention of the characteristic proteins present in the membrane of these cancer cells. The results are further supported by the cell culture internalization study (see below).

### II.D MRI Properties Depend on NPs Clustering

**Figure 4:**
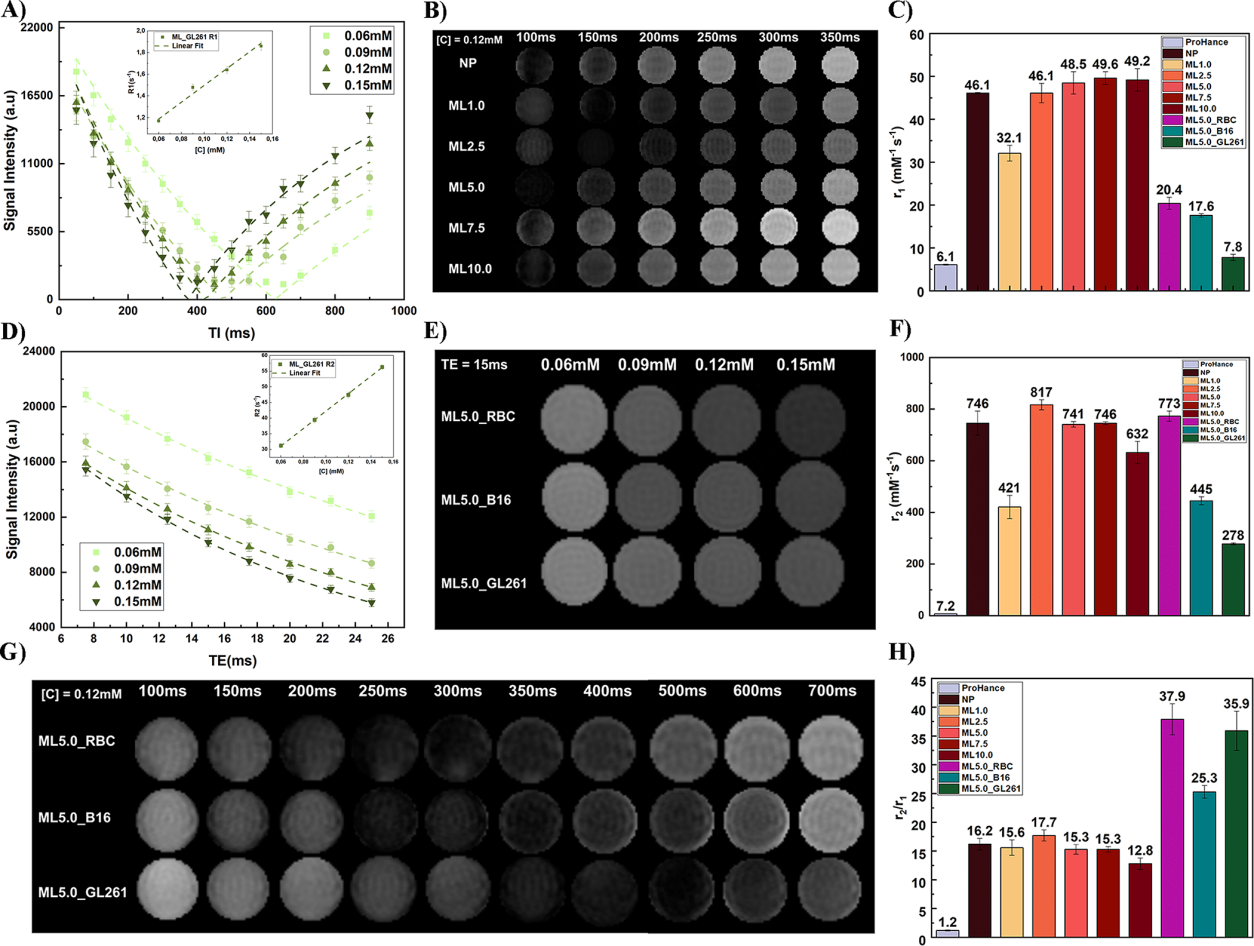
(A) MRI inversion recovery (IR) data for the T1 determination of sample ML_GL261. The inset shows the relaxivity concentration dependence. (B) Representative TI images for NP and synthetic ML samples with [Fe] + [Mn] = 0.12 mM. (C) *r*_1_ for all samples. (D) MRI spin echo (SE) data for the T_2_ determination of sample ML_GL261. The inset shows the relaxivity concentration dependence. (E) Representative T2 images for hybrid ML samples with spin echo time TE = 15 ms. (F) *r*_2_ for all samples. (G) Representative TI images for all hybrid samples at [Fe] + [Mn] = 0.12 mM. (H) *r*_2_/*r*_1_ values for all samples.

After the detailed NP characterization, we evaluated the MRI contrast agent properties. Figure 4(A) shows the MRI signal intensity as a function of distinct inversion times for the hybrid glioblastoma magnetic vesicle, ML5.0_GL261. Symbols represent experimental data at distinct concentrations, while the line is the best fit using eq 1
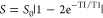
1

From each curve, it is possible to extract *T*_1_. The inset of Figure 4(A) shows the analysis of 1/*T*_1_ as a function of particle concentration. The linear fit reveals the longitudinal relaxivity *r*_1_, that for this sample was found to be 7.8 mM^–1^ s^–1^. Similar analysis was performed for all other samples, including the control ProHance. Figure 4(B) shows typical images obtained for all of the synthetic samples evaluated, while Figure 4(C) shows the value obtained for *r*_1_. For ProHance we obtained 6.1 mM^–1^ s^–1^, while for the citrate-coated NPs we found 46.1 mM^–1^ s^–1^. ML1.0 showed a slight decrease to 32.1 mM^–1^ s^–1^, increasing to 49.2 mM^–1^ s^–1^ for ML10.0.

The analysis of *T*_2_ used a spin echo sequence, where the signal intensity is fitted with the following equation
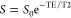
2Figure 4(D) shows the signal intensity as a function of spin–echo time (TE) for the sample ML5.0_GL261. Symbols represent experimental data at distinct particle concentrations, while the line is the best fit using eq 2. The inset shows 1/*T*_2_ as a function of particle concentration. Note, as expected, the linear concentration dependence. From this sample, we obtained the transverse relaxivity r_2_ of 278 mM^–1^s^–1^. Figure 4(E) shows images of all hybrid samples considering a spin echo time of 15 ms (results for the synthetic MLs can be found in SI, Figure S4). Figure 4(F) shows the *r*_2_ values for all samples. For ProHance we found 7.2 mM^–1^s^–1^, while for the citrate-coated NPs, we found 746 mM^–1^s^–1^. The value decreases to 421 mM^–1^s^–1^ for ML1.0 and then increases, achieves a maximum for ML2.5, decreasing to 632 mM^–1^s^–1^ for ML10.0. For the hybrid samples, we found 773 mM^–1^s^–1^ for ML5.0_RBC, 445 mM^–1^s^–1^ for MLB16, and 278 mM^–1^s^–1^ for ML5.0_GL261.

Figure 4(G) shows typical inversion time images of the hybrid vesicles. It is clear the difference in *r*_1_. For ML5.0_RBC we obtained 20.4 mM^–1^ s^–1^, while for MLB16 we found 17.6 mM^–1^ s^–1^, decreasing to 7.8 mM^–1^ s^–1^ for ML5.0_GL261. The longitudinal relaxivity decreased for the larger hydrodynamic sizes but seems to depend on other factors. For instance, for the hybrid samples, the highest value is found for ML5.0_RBC, followed by MLB16 and ML5.0_GL261. Curiously, the ESR line width data showed similar behavior.

The ratio of *r*_2_/*r*_1_ is shown in Figure 4(H). For ProHance we found a value of 1.18, that is similar to previous analysis of other groups that found 1.19 for 0.47 T and 1.10 for 1.5 T at 37 °C.^50^ The ratio for the Mn-ferrite NP was 16, revealing that the NP at this size is an excellent T2 contrast agent. Again, similar results were reported in the literature. Leal et al. found 21 for monodisperse Mn-ferrite samples of 14 nm using a 1.5T MRI equipment.^51^ For the MLs we found that encapsulation of NPs inside the vesicles resulted first in a slight increase in comparison to the magnetic fluid for ML2.5 (from 16 to around 18), and then a decrease for samples ML5.0, ML7.5, and ML10.0, the latter with a value close to 13. T2 contrast agents are established for *r*_2_/*r*_1_ values higher than 10, while *r*_2_/*r*_1_ lower than 5 are observed for T1 agents. The decrease of the ratio the higher the encapsulation suggests that one might enhance the *r*_1_ properties through packing/clustering. Therefore, it might be possible to expect that decreasing the core diameter, could result in dual contrast agent properties. In the contrary, the hybrid vesicles showed an even higher ratio value. For ML5.0_RBC *r*_2_/*r*_1_ equals 38, followed by ML5.0_GL261, 36, decreasing to 25 for MLB16. All the results are higher than the NP and synthetic MLs, and indicate a better T2 contrast agent.

The *r*_2_ values reported in this study are high in comparison to several studies in the literature,^52^ but similar results have been reported before.^3^ One might explain it due to the high saturation magnetization of Mn-ferrite NPs, the large particle size and also the multicore NP organization.^3^ Indeed, clustering magnetic NPs can increase the relaxivity, as demonstrated by Zhou et al.^53^ NP clustering can be relevant because it induces distinct stray field gradients from aggregate formation, which impacts *r*_2_. The core diameter (15 nm) suggest that the NP should be governed by the outer sphere model, also named as motional averaging regime (MAR).^52^ In this case, increasing the NP volume fraction should result in higher relaxivity values. Higher particle volume fraction inside the liposome can affect the diffusion correlation time, probably increasing the viscosity inside the vesicle.^52^ Again one would expect an increase of *r*_2_. Our results point into the other direction, since liposomes with higher hydrodynamic sizes showed lower values. One might argue that clustering could induce a transition from MAR to the static dephasing regime (SDR) and then to the partial refocusing regime (PRR), resulting in the appearance of maximum and decrease of r_2_.^52^ PRR scales inversely with the diffusion correlation time, such that an increase in viscosity results in a decrease of the relaxivity.^52^ On the other hand, the aggregate shape inside the vesicles could depend on the ML hydration step (previous magnetic fluid concentration). At higher concentrations, even at the MAR regime, one could imagine that more spherical-like shaped clusters are formed, which could decrease the field gradient generated by those clusters and explain the data. The shape of the NPs has a huge effect in MR properties, for instance Zhao et al, found very high relaxivity values for octapods in comparison to spherical NPs.^54^ Overall, our results suggest that clustering governs the MRI properties of the vesicles.

### II.E *In Vitro* Experiments Suggests MLs Uptake and Thermal-Induced Cell Death

**Figure 5:**
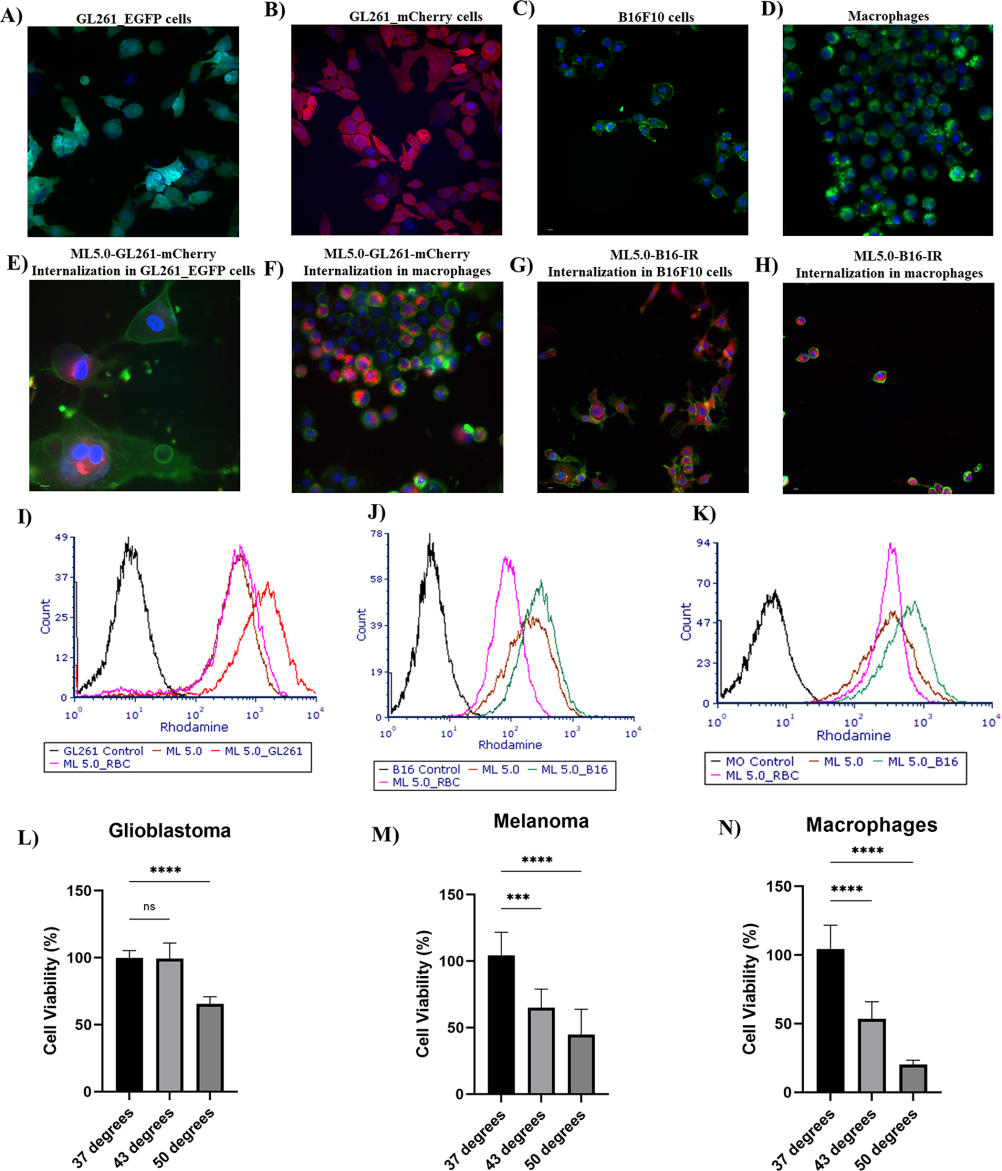
Immunofluorescence images acquired using a CellInsight CX7 LZR Pro (ThermoFisher). Cell membranes were labeled with Wheat Germ Agglutinin – Alexa Fluor 488 (green) and the nuclei with Hoechst 33342 (blue). The red fluorescence was used for the identification of GL261-mCherry fragments autofluorescence or for formulations labeled with IR780. GL261-eGFP autofluorescence was also identified by green. (A) and (B) Glioblastoma GL261-eGFP and GL261-mCherry cell lines, respectively. (C) B16F10 melanoma cells. (D) macrophage J774A.1 cells. (E) and (F) merged images showing MLs-GL261-mCherry upatake by GBL and macrophages cells, respectively. (G) and (H) merged images showing the uptake of MLs hybridized with B16F10 membranes by melanoma and macrophages cells, respectively. (I)-(K) Cellular uptake of nanocarriers by GL261, B16F10 and J774A.1 cells, respectively, evaluated by flow cytometry. (L)-(N) Thermal bath cell viability study. Cell viability was calculated in relation to control samples incubated at 37 °C with 5% CO_2_.

The biomimetic MLs developed are expected to also be used as heat generating centers during PTT or MNH therapies. Therefore, we evaluated MLs-cell interactions by immunofluorescence images and the nanoparticles uptake are presented in Figure 5. Melanoma (B16F10) and macrophage (J774A.1) cell lines had their cell membranes labeled by WGA (green), whereas glioblastoma cell lines GL261-eGFP and GL261-mCherry present autofluorescence in green and red, respectively (Figure 5(A)–(D)). Cell nuclei were stained with Hoechst 33342 (blue). Panel (E) shows a merged image confirming ML5.0_GL261-mCherry uptake by GL261-eGFP cell line, whereas Panel (G) shows ML5.0_B16F10 uptake by B16F10 melanoma cells. While hybrid ML5.0_B16F10 liposomes were labeled with IR780 for visualization, hybrid ML5.0_GL261-mCherry derived vesicles internalization could be visualized by their autofluorescence in 561 nm. This feature highlights the potential of biomimetic ML5.0_GL261 as a theranostic nanocarrier once it did not need additional labeling for fluorescent microscopy detection. Additionally, uptake experiments using macrophages were performed for comparison. As can be seen on Panels (F) and (H), both GL261-mCherry and B16F10 biomimetic liposomes were internalized by J774A.1 cells, as expected. Complementary studies of cellular uptake were also performed using flow cytometry. As can be seen in Panels (I)–(K), our results suggest differential uptake dependence of biomimetic nanocarriers regarding the plasmatic membrane that they were derived, i.e. ML-GL261 liposomes were preferentially internalized by glioblastoma and ML-B16F10 liposomes by melanoma cells, after 4 h of exposure. Furthermore, no substantial differences were verified over the nanocarriers uptake by J774A.1 macrophages. Notabily ML-RBC liposomes presented lower internalization profile for the 3 cells lines tested, which could be in accordance with the camouflage and long time circulation features associated with RBC-coated nanoparticles previously reported, probably due to the high content of CD47 in RBC membranes, a protein that inhibits internalization.^8^

Finally, to evaluate cell lines’ thermal sensibility, MTT was used to quantify cell viability toward heat bath (Panels (L)–(M)). Temperature-induced cytotoxicity was verified, since the number of viable cells in control samples (37 °C) remained unchanged over time, whereas for distinct temperatures, 43 and 50 °C for 15 min, a significant decrease in cell viability in a thermal dose manner could be observed for the 3 cell lines tested. This suggests that if MLs could deliver these thermal doses, one would verify cell death. The results are in agreement with those reported by our group elsewhere.^41,42^ It is worth noting that neither magnetic particles nor MLs were capable of decreasing the cell viability of glioblastoma and melanoma cells for all concentrations used in this work (Figure S5). This is indicative that any decrease in cell viability is related with the suggested therapy.

### II.F Magnetic Hybrid Vesicles Are Powerful Thermal Agents

**Figure 6:**
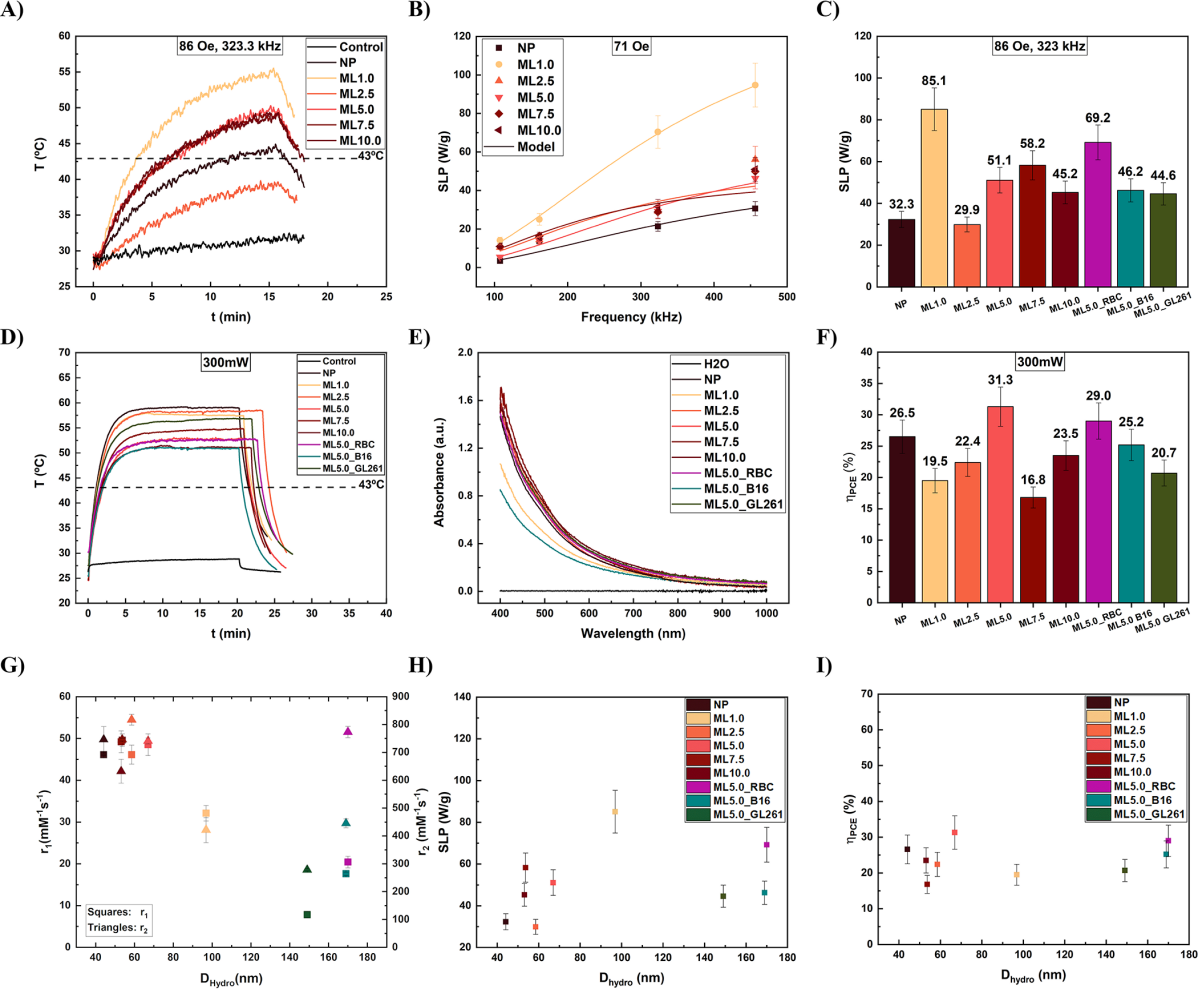
(A) MNH temperature profile during MNH, at 323 kHz and 86 Oe. (B) MNH frequency study at 71 Oe and distinct AC field frequencies. Symbols represent data, while lines are the fit using the LRT model. (C) MNH efficiency, SLP, for distinct samples, at 323 kHz and 86 Oe. (D) PTT temperature profile for distinct samples. (E) Absorbance curve of the samples. (F) Photothermal conversion efficiency (PCE) values of the samples. (G) Relaxivity hydrodynamic size dependence. (H) SLP hydrodynamic size dependence. (I) PCE hydrodynamic size dependence.

To verify the heat generation properties of the MLs detailed *in vitro* experiments were performed. Figure 6(A) shows the temperature profile of several samples for MNH, considering a frequency of 323 kHz and a field amplitude of 86 Oe (6.8 kA/m). The dashed line represents the therapeutic temperature of 43 °C. The best result was found for ML1.0, and as expected, no significant heat arises from the control sample (water). Figure 6(B) shows the heating efficiency (SLP) as a function of magnetic field frequency for a fixed field amplitude (71 Oe). Symbols represent distinct samples, while the solid line is the best fit using the linear response model. LRT seems to represent well the data because of the low field conditions. From this analysis we are able to extract the equilibrium susceptibility and the relaxation time responsible for heat generation. Table S1 shows the two fitting parameters extracted from this analysis. The effective magnetic relaxation time was found to be on the order of 10^–7^ s. Similarly as a previous study of the group with albumin-coated Mn-ferrite nanocarriers,^31^ the single particle relaxation, Brownian or Néel, does not explain the data. Indeed, the heating efficiency seems to be related to collective magnetic relaxation.^31^Figure 6(C) shows the estimated SLP values for the samples investigated at the highest field condition of 323 kHz. The best samples was ML1.0 for this experimental situation. However, the values are competitive for such low field and clinically relevant condition.

The PTT study is reported in Figure 6(D), (E), and (F). Note that the temperatures achieved are higher than the ones obtained for MNH, suggesting a more efficient heat generation mechanism for PTT, under this condition. Control showed almost no heat generation, while all samples achieved temperatures in the therapeutic regime. Photothermal stability study is reported in SI for the hybrid vesicles (Figure S6), confirming no photodegradation and the excellent heating perfomance of the nanocarriers under multiple irradiations. PTT efficiency correlates with the absorption coefficient at the laser wavelength (808 nm). Figure 6(E) shows the absorbance curves for all of the samples. As expected for ferrite NPs, the absorption increase for lower wavelengths. At the near-infrared region it decreases considerably, however the samples are still very efficient as photothermal agents. Indeed, we used the Roper’s method to determine the photothermal conversion efficiency (PCE), that measures the amount of light that is converted into heat. Figure 6(F) shows the PCE values obtained for the MLs, which are ∼25–30%. These values are lower than some reported for gold nanostructures, but very competitive. In addition, different from gold nanostructures, iron oxide based nanoparticles as well as liposomes are well established in the clinic.^55^

Furthermore, we investigated the role of hydrodynamic size in the theranostic properties. Figure 6(G), (H) and (I) show respectively the MRI relaxivities (*r*_1_, *r*_2_), the MNH efficiency, and the PTT light–heat conversion values as a function of the hydrodynamic size. Both *r*_1_ and *r*_2_ showed decreases for higher sizes. As discussed before this could be related to a transition due to particle aggregation from MAR to the static dephasing regime (SDR) and then to the partial refocusing regime (PRR). Figure 6(H) suggests an optimum SLP value of around 100 nm. This behavior is consistent with a previous study of the group using albumin-based magnetic nanocarriers, suggesting that magnetic Néel collective relaxation is responsible for MNH heat generation.^31^ On the other hand, the PCE values showed no clear size dependence. This is curious since classical electromagnetic theory, namely Mie’s theory, suggest that larger sizes would show higher scattering effects, which as a result decrease light absorption. It seems that this might not be highly relevant at these conditions, suggesting that aggregation effects observed during nanoparticle internalization into the cells might not decrease the photothermal efficiency. The same argument might not be true for MNH,^56^ suggesting an advantage for PTT if a tumor location is achievable by fiber optic.

### II.G Theranostic Applications with Biomaterial

After the cell culture evaluation and thermal properties evaluation for the MLs, we designed a proof-of-concept set of experiments to demonstrate near-infrared imaging, magnetic resonance imaging, and photothermal and magnetic hyperthermia properties. The near-infrared dye IR780 is incorporated in the membrane of the magnetoliposomes of ML5.0, named as ML5.0_IR780. The IR780 concentration is 150 μg/mL, while the magnetic NP concentration is 5 mg/mL. The *ex vivo* biological material consists of a pork loin, as similarly used by others.^57^

**Figure 7:**
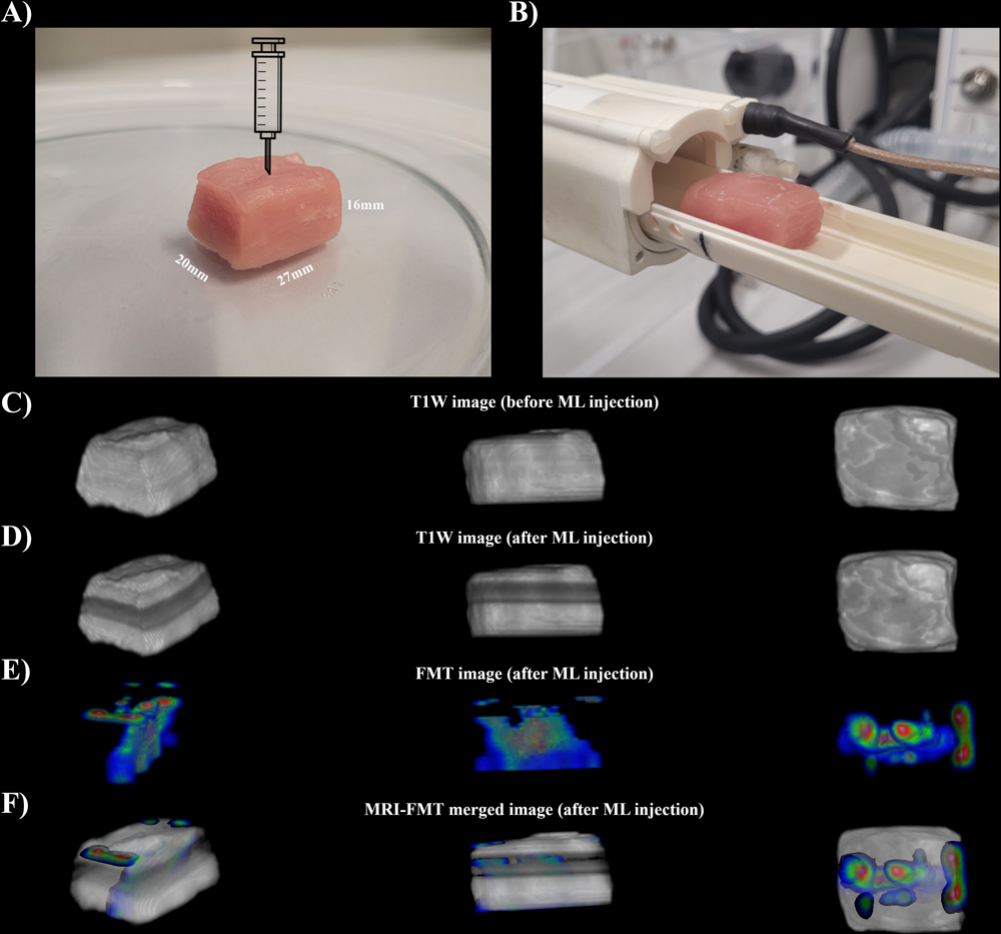
(A) Pork loin dimensions used in the *ex vivo* experiment. ML containing IR780 dyes in the membrane is injected 5 mm below the surface. (B) Image of the position of the pork loin in the MRI RF coil. (C) T1W MRI image before ML injection. (D) T1W MRI image after ML administration. (E) FMT image of the near-infrared ML inside the pork loin. (F) MRI-FMT merged image.

Figure 7(A) shows the dimensions of the pork loin. The loin is positioned inside the radio frequency (RF) coil (Figure 7(B)). MRI T1W images are obtained for the biological material before (Figure 7(C)) and after (Figure 7(D)) ML administration. 120 μL of ML5.0_IR780 is injected 5 mm below the surface of the loin. It is clear from the image that the NPs promote a contrast in the MRI image.

Figure 7(E) shows the 3D reconstruction of the luminescent magnetoliposome inside the biomaterial using fluorescence molecular tomography (FMT). The channel 790 nm was used for excitation. The fluorescent ML is shown to distribute around the injection site in a nonuniform way. Figure 7(F) shows the merged MRI-FMT 3D image of the biomaterial investigated. In the Supporting Information, we include a video showing the 3D images at different positions. The results indicate that the multifunctional vesicle has interesting imaging applications.

**Figure 8:**
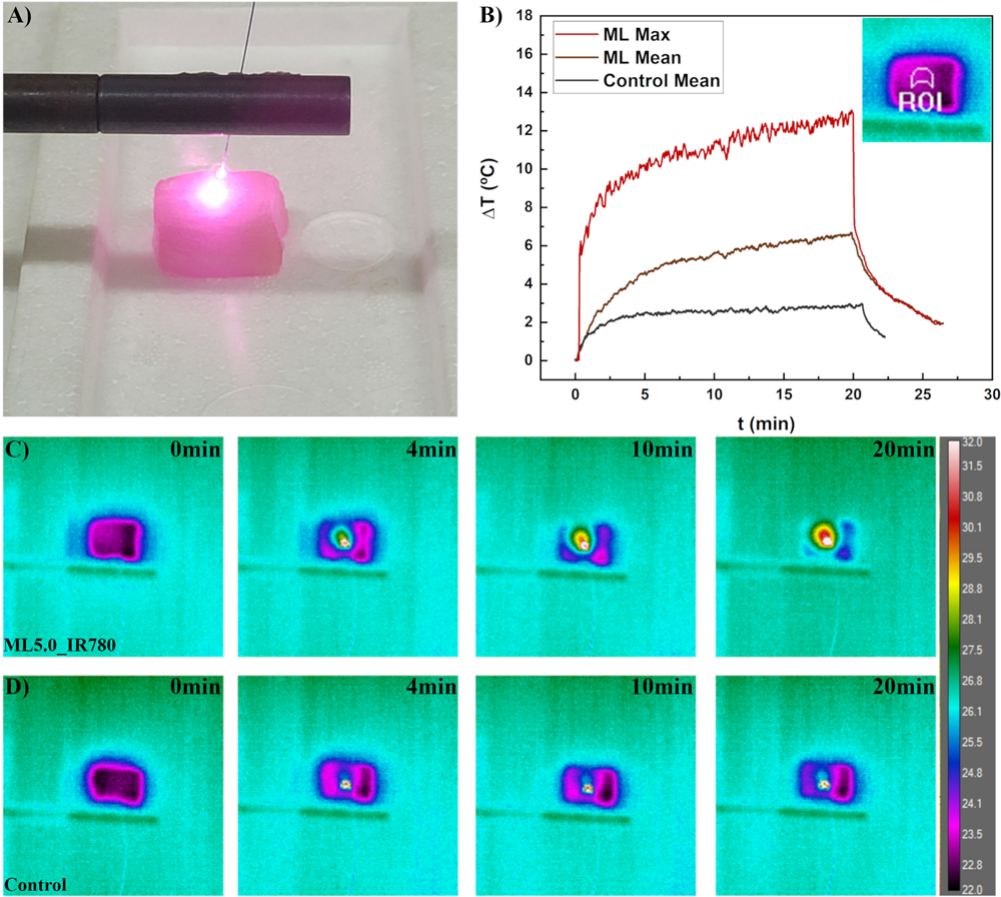
(A) Image of the PTT experiment in the pork loin containing MLs with IR780 incorporated in the membrane, sample ML5.0_IR780. (B) Mean temperature profiles considering the same ROI for samples without MLs (control) and with ML5.0_IR780 mean and maximum temperature profiles. Thermal camera snapshot images at distinct PTT times for (C) ML5.0_IR780, and (D) control.

Figure 8(A) shows an image of the biomaterial during PTT. The image was obtained with a cell phone that is able to detect near-infrared light that arises due to the IR780 dyes (pink color in the loin). From each image we choose a ROI for monitoring the mean temperature with a thermal camera. The inset of Figure 8(B) shows the ROI designed for the experiments. The PTT temperature profile data of a control sample injected with water (120 μL) in another pork loin, as well as the biomaterial with ML5.0_IR780 is shown in Figure 8(B). The thermal camera evaluates the temperature at the surface of the biomaterial. This proves that the NPs are heating due to the photothermal effect. Figure 8(C) and Figure 8(D) show thermal camera snapshots of both samples at distinct PTT times of treatment. For this experimental setup we found a temperature variation for the control sample of around 2 degrees, while the one containing the MLs varied by more than 7 degrees. However, within the ROI, maximum temperatures as high as 12 degrees are reported. Since thermal camera only monitors surface temperature, it is obvious that far higher temperatures are achieved during PTT. For instance, similar experiments using luminescent nanothermometers prove higher intrabiomaterial temperature.^58^ Similar study was performed with MNH (Figure S7). MNH showed lower temperature variation because of a lower efficiency in comparison with that of PTT.

### II.H Intratumoral Nanoparticle Administration Demonstrates NP-Mediated PTT

As a proof of concept, some *in vivo* experiments were performed using the B16F10 murine tumor model. We explored three distinct routes of nanoparticle administration, intratumoral (I.T.), intravenous (I.V.) and intraperitoneal (I.P.). Control animals, with similar tumor volume, were used for comparison with the animal that received the biomimetic NP, namely MLB16_IR780. New biomimetic nanoparticles were prepared increasing the number of freeze and thaw cycles to obtain hybrid liposomes. Figure S8 shows the hydrodynamic size and PDI results of the hybrid vesicles. Note that PDI decreased to around 0.2 due to the increase of cycles, as expected. For instance, the MLB16_IR780 showed a size of 143 nm and PDI of 0.26.

The first *in vivo* study consisted of the I.T. administration of the biomimetic magnetoliposome in one animal. Figure S9(A) shows MRI images of pre and post NP administration. The NP effect is clearly observed comparing both T1w images. Similarly, fluorescence molecular tomography confirms the NP I.T. administration. Figure S9(B) shows representative thermal images obtained during the PTT study, at low laser power condition, 150 mW. On the top the results correspond to the preadministration (control) case, while on the bottom it is shown the postadministration. The temperature profile is shown in Figure S9(C), where before NP injection the animal’s surface tumor temperature achieved around 40 °C, while after I.T. administration one observes around 45 °C. The results clearly demonstrate the nanoparticle-mediated PTT effect.

### II.I Intravenous Nanoparticle Administration Supports MRI-Guided PTT

**Figure 9:**
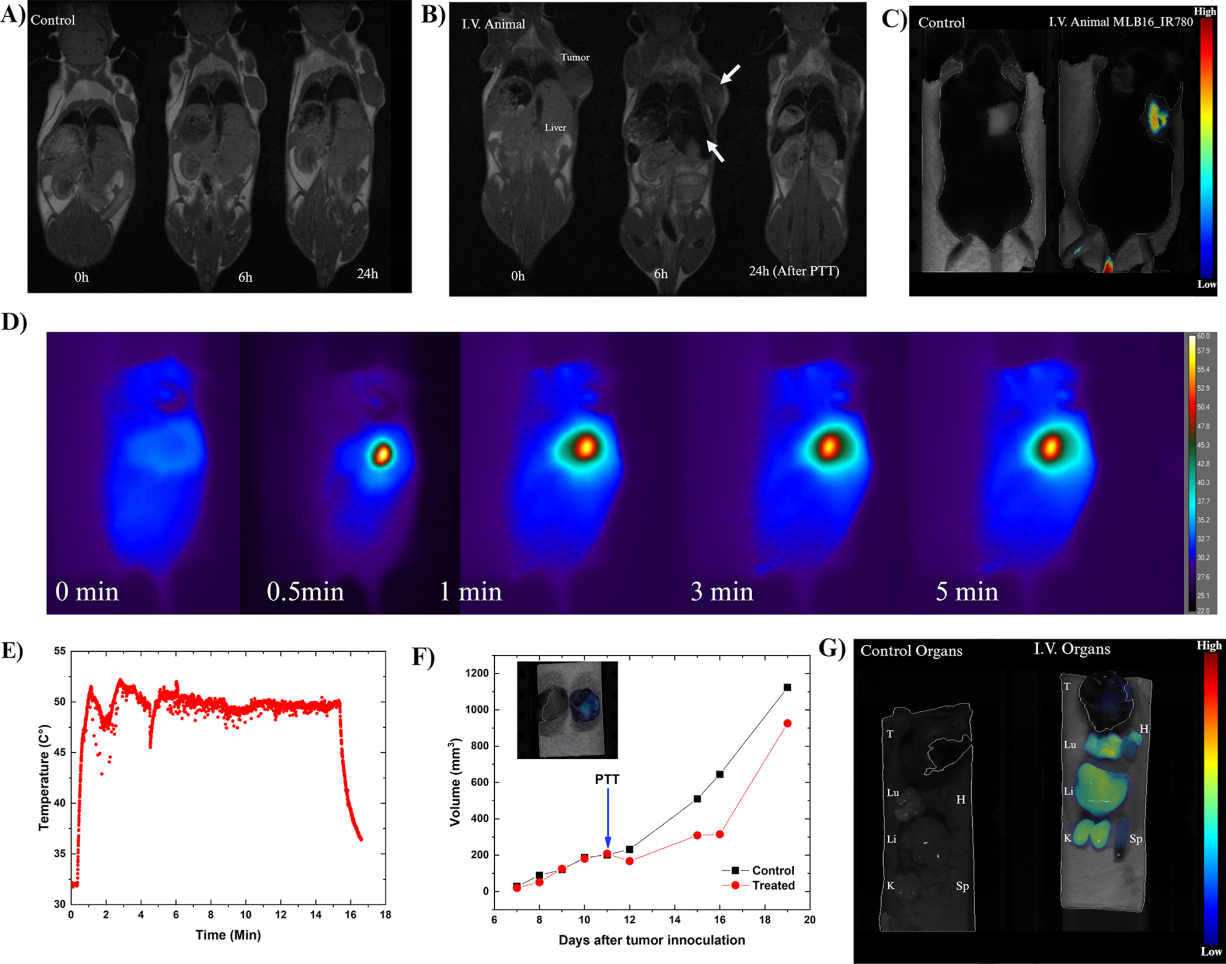
(A) T1W MRI images of the control animal at distinct times (0, 6, and 24 h). (B) T1W MRI images of an animal that received intravenous (I.V.) administration of MLB16_IR780 at distinct times (0, 6, and 24 h). The image of the I.V. animal at 24h corresponds to a post PTT procedure. (C) FMT images of both animals, 24h after I.V. administration, before PTT. (D) Thermal images of animal I.V., 11 days after tumor inoculation, considering distinct times of PTT. (E) Mean temperature profile during PTT for a ROI located on the tumor. (F) Tumor growth of both control and I.V. animals. The arrow indicates the day of PTT. The inset shows FMT images of both animals’ tumors, 19 days after innoculation. (G) FMT images showing the biodistribution of MLB16_IR780, 19 days after tumor innoculation (8 days after PTT).

The other experiment performed was the I.V. study. In this case, we separate two animals with similar tumor volumes and followed them for several days. Figure 9(A) and (B) show T1w MRI images of the control and I.V. animals at distinct times after NP administration. It is clear in Figure 9(B) the success of NP I.V. administration, since in both liver and tumor one can observe the signature of NPs (see the arrows), 6h after NP administration. In the liver, one can easily observe a darker image in the organ due to the high amount of magnetic NPs, while in the tumor, one can note a black dot that is indicative of NP accumulation. Figure 9(C) shows the FMT images of both animals, as expected no fluorescence in the control animal and a signature of near-infrared images in the tumor and the tail for the other one, that further indicates that there might be NPs in blood circulation even 24 h after administration. The FMT image was taken before PTT. Figure 9(D) shows representative thermal images of the treated animal, while Figure 9(E) indicates the mean temperature profile considering a ROI around the laser irradiation area. For the PTT procedure, we tuned the laser power for not allowing temperatures higher than 50 °C, and established 15 min of treatment. Some temperature variations are observed due to some animal movement during the procedure, and tuning of the laser power to maintain thermal dose.

Figure 9(F) shows the tumor growth profiles for both animals. PTT happened 11 days after tumor innoculation (see arrow). The control animal was chosen because of similar tumor growth. MRI image after PTT is shown in Figure 9(B). One can clearly observe the effect of PTT treatment on the tumor. The same is said in the tumor growth profile that showed that the control animal tumor volume increased faster than that of the PTT treated animal. After 19 days, the animals were euthanized. The inset shows the FMT image of both tumors, control, and PTT treated animal. Figure 9(G) shows the FMT biodistribution images of the organs for both animals. One can see the near-infrared biomimetic NP signal at several organs, including in the heart, that suggests that there were still biomimetic NPs in circulation. Figure S10 shows the GRE MRI images of the same organs that support similar conclusions. The I.V. animal clearly shows evindence of a high concentration of NPs in some spots of the liver, lung, and tumor, while a darker image of the spleen suggests a great concentration in the whole organ.

### II.J Intraperitoneal Nanoparticle Administration Also Indicates Nanoparticle Tumor Accumulation

Finally, we investigate the biodistribution and NP tumor accumulation by intraperitoneal administration. Figure S11 shows T1w MRI images of the animal administered I.P. with MLB16_IR780, at distinct times. Two slices are shown to better observe organs and tumor. In this case, a higher amount of NP is injected and the NP effect on the images is used to better identify the NP trafficking, as indicated by arrows. Distortions in the image are mainly due to the high magnetic NP content. We observed NP effect in expected regions as liver, lungs, and later in the spleen, but curiously images at 6h revealed a high content of NPs in the thymus. The trafficking might be related to the delivery of NPs by macrophages that could have internalized them in the I.P. region. The post administration image at 0h also suggests that few NPs after I.P. administration might have arrived to the liver by the blood circulation system.

After several days, the control and I.P. animals were euthanized for biodistribution study. Figure S12(A) and (B) shows the GRE MRI and FMT images of the control and I.P. animals, respectively. The FMT image shows NPs in several organs of the animal with I.P. NP administration. GRE MRI images also indicate NP accumulation. Indeed, to further check the efect of NP in this sequence an *in vitro* study with the magnetic nanoparticles were performed to evaluate how NP concentration affects the GRE image. Figure S12(C) shows the GRE results, indicating that low particle concentration results in a brighter image, while increasing NP concentration makes the image darker and promotes distortions in the image. The liver in Figure S12(B) is far darker than the control (see Figure S12(A) due to high content of magnetic NP. Meanwhile, comparison of the other organs of both animals suggests that the brighter images in the tumor, lung, heart, and spleen indicates a small amount of NPs in those organs.

Figure S12(D) shows only the FMT images of the tumor, which in this scale clearly indicate NP intratumoral accumulation after I.P. administration. Similar results have been reported before by Toraya-Brown et al.,^59^ which suggests that the trafficking to the tumor was due to macrophages, after endocytosis of the magnetic NPs in the I.P. region. Furthermore, in both I.P. and I.V. studies, the GRE MRI images of the heart suggest that there are magnetic biomimetic NPs in blood circulation, even after several days of administration. All of the results suggest that monitoring the tumor with MRI can guide the best moment for PTT treatment.

## III Conclusions

In this work, we prepared hybrid biomimetic magnetic vesicles for theranostics. The encapsulation of the Mn-ferrite nanoparticles into the vesicles depends on the hydration step, magnetic fluid particle concentration, and the extrusion/sonication process. Hybrid liposomes consisted of a mixture of extrinsic lipids and cell membrane fragment bilayers enclosing magnetic nanoparticles. Results with three different plasmatic membranes are reported, namely red blood cells (RBC), melanoma (B16F10) and glioblastoma (GL261). RBC coating has been reported in the literature, but as far as we know, this is the first demonstration of biomimetic magnetoliposomes with B16F10 or GL261 membrane fragments. After the MLs preparation, the near-infrared dye IR780 was incorporated into the membranes for NIR imaging, allowing three-dimensional monitoring using fluorescence molecular tomography. GL261 expressing fluorescent protein mCherry was used for hybridization of GBM vesicles. Immunofluorescence confirmed the success of the biomimetic nanocarriers, and flow cytometry results support differential wrapping times depending on cell membrane fragment from which the biomimetic vesicles were derived. Detailed MRI studies revealed the longitudinal and transverse relaxivity properties of the MLs. Ultrahigh *r*_2_ value is reported for the Mn-ferrite nanoparticles due to the size and multicore nanostructures. Hybrid magnetic vesicles showed lower *r*_2_ values but strong T2 contrast agent properties. Both thermal therapy modalities, MNH and PTT, achieved therapeutic temperatures, but PTT was found to be more efficient than MNH for the *in vitro* conditions studied. Competitive values for the photothermal conversion efficiency (PCE) are obtained, suggesting that this nanomaterial has great potential for thermal therapy clinical studies. Although the PCE value is lower than gold nanostructures, the use of iron oxide based NPs and liposomes in the clinic strongly suggests its biomedical potential. Furthermore, *ex vivo* experiments, using pork loin, were first performed to demonstrate the theranostic applications of the magnetic vesicles. Finally, complementary *in vivo* experiments revealed NPs tumor accumulation over three different administration routes, achievement of clinical temperatures, and retardation of tumor growth, indicating that these nanocarriers could be used for MRI-guided thermal therapy.

## IV Materials and Methods

### IV.A Chemicals

Manganese(II) chloride tetrahydrate (MnCl_2_·4H_2_O), iron(III) chloride hexahydrate (FeCl_3_·6H_2_O), and iron(III) nitrate nonahydrate (Fe(NO_3_)_3_·9H_2_O) were purchased from Vetec (Rio de Janeiro, Brazil). Nitric acid (HNO_3_), acetone, and sodium citrate tribasic dihydrate (Na_3_C_6_H_5_O_7_·2H_2_O) were purchased from Cromoline (Diadema, Brazil). Hydrochloric acid (HCl) was purchased from Qhemis (Jundiaií, Brazil). Soy phosphatidylcholine (SPC, Lipoid S100) was purchased from Lipoid GmbH (Ludwigshafen, Germany); cholesterol (CHOL), Wheat Germ Agglutinin-Alexa Fluor 488 (WGA), Hoechst 33342 and IR780 iodide were from Merck (Darmstadt, Germany), and 1,2-dioleoyl-*sn*-glycero-3-phosphoethanolamine-N-(lissamine rhodamine B sulfonyl) (Liss Rhod PE) was purchased from Avanti Polar Lipids (Alabaster, USA). Cell culture reagents such as Dulbecco’s Modified Eagle’s Medium, fetal bovine serum, and penicillin–streptomycin were purchased from ThermoFisher Scientific (Waltham, MA, USA). Propidium iodide (PI) and MTT assay kit were purchased from Sigma-Aldrich (Missouri,USA). All other reagents were purchased from Merck (Darmstadt, Germany) or ThermoFisher Scientific (Waltham, MA, USA) at the highest available purity grades.

### IV.B Magnetoliposomes Preparation

The magnetic nanoparticles synthesis was via coprecipitation method, as described in detail in a previous work^29^ and in the Supporting Information.

In this article, SPC vesicles enriched with cholesterol were used to derive magnetoliposomes (ML) and three different magneto cell-membrane-hybrid liposomes composed of preformed SPC bilayers fused with membrane fragments, namely, ML-B16 (containing B16–F10 murine melanoma membrane fragments), ML-GL (containing GL-261 murine glioblastoma membrane fragments), and ML5.0_RBC (containing erythrocyte membrane fragments).

SPC vesicles (20 mM) were prepared by dissolving soy phosphatidylcholine and cholesterol (30 mol %) in chloroform to prepare a film of lipids by rotary evaporation under pressure (IKA RV 10). Subsequently, the films were hydrated using the ferrite nanoparticle colloidal suspension in the desired magnetic concentration to form multilamellar liposomal suspensions. Unilamellar liposomes were obtained after rigorous extrusion using polycarbonate filters with 0.2 μm diameter pores or by sonication.

Next, SPC liposomes were added to cell membrane fragment solutions, followed by 50 freeze–thaw cycles to obtain hybrid liposomes. Erythrocyte membranes were isolated by hypotonic lysis following a previously described protocol.^16,41^ Briefly, human blood, obtained from blood banks, was diluted in a phosphate saline buffer (PBS, 10 mM phosphate, 154 mM NaCl, pH = 7.4) and centrifuged at 150 × *g* during 10 min at 4 °C to isolate the RBC. After that, the plasma and white blood cells were carefully removed by aspiration in three different cycles and RBCs were diluted in a lysis buffer solution (5 mM of phosphate, pH = 8.0) for 12 h at 4 °C and subsequently centrifuged at 25000 × *g* for 10 min at 4 °C several times to remove the residual hemoglobin molecules. Melanoma and glioblastoma membranes were obtained after B16F10 or GL261 cell disruption by repeated freezing and thawing cycles followed by differential centrifugation for the isolation of membrane fragments, as described in ref (41). The total membrane protein content of samples was determined using a commercial kit (Sigma-Aldrich, Missouri, USA) based on the reaction of bicinchoninic acid (BCA). Finally, the final suspensions containing hybrid liposomes at the 4:1 (w/w) SPC:cell-membrane ratio were rigorously extruded by using polycarbonate filters with 0.2 μm diameter pores or sonicated to form unilamellar vesicles.

IR780 were incorporated into liposomes by a postinsertion protocol. A stock solution containing the dyes diluted in chloroform were used to prepare thin films in the bottom of glass tubes under a gaseous nitrogen flow. The vesicles were placed in contact with films under slow orbital shaking (IKA, KS 400) for 30 min, and the excess dye was removed by filtration.

### IV.C Biophysical Characterization

Biophysical characterization of all ML nanocarriers was performed, and their morphology, NP content, concentration, size, and superficial charge were determined. For each ML, morphology was assessed by transmission electron microscopy (TEM) using a JEOL JEM-2100 microscope (Tokyo, JP). The ML samples were fixed using a 25% (v/v pH 7.0) buffered formaldehyde solution and subsequently postfixed with an osmium tetroxide solution (4% v/v). Finally, ML samples were dehydrated in ethanol, deposited on the carbon film of a TEM copper grid, and colored with 0.5% aqueous uranyl acetate. For determination of NP content, MLs from different concentrations were selected, and their total area as well as the NP aggregates area were manually delimitated via ImageJ software. The LUT Spectrum from ImageJ was applied to the TEM images to enable better edge detection of both liposomes and their NP aggregates content.

MLs concentration and size were obtained by nanoparticle-tracking analysis (NTA, NanoSight NS500 equipped with a 532 nm laser and an EMCCD 215S camera, NanoSight, Amesbury, UK). Vesicles suspensions were diluted in a ratio of 1:10^5^ before analysis and automatically injected into the sample cell. Each liposome concentration and size distribution were obtained using the NTA 3.4 software. Zeta-potential measurements were performed using a Zetasizer Nano ZS90 (Malvern Panalytical, Westborough, MA, USA).

Each ML IR780 concentration was quantified by emission measurements performed in a Horiba-Jobin Yvon spectrofluorimeter (Tokyo, Japan), Model Fluorolog-3 (FL3–221), under excitation with an external laser source of 804 nm (80 mW) connected to a Spectracq2 data acquisition module and an R5509-73 PMT InGaAs detector. In addition, MLs absorption curves were recorded in the 400–1000 nm wavelength range at room temperature using a Cary 50 UV–vis spectrophotometer (Varian Inc., Palo Alto, CA, USA) equipped with a full-spectrum Xe pulse lamp single source. Samples were diluted to prevent light scattering in both experiments.

For protein characterization of each ML, denaturing SDS-PAGE was performed according to reference.^200^ In brief, 7.5 μL of membrane fragments and cell-membrane-derived vesicles (5–25 μg protein) were mixed with 2.5 μL of Invitrogen sample loading buffer and heated at 70 °C for 10 min. The samples were then run on a NuPAGE Novex 4–12% Bis-Tris minigel (Invitrogen) with 5 μL of prestained SDS-PAGE Standards (Bio-Rad) loaded. Finally, electrophoresis was performed at room temperature for approximately 45 min using a constant voltage (200 V) in a solution of NuPAGE MOPS SDS Invitrogen running buffer until the dye front reached the end of the 60 mm gel.

### IV.D Magnetic Characterization

Room-temperature magnetization data were obtained using a VSM model EV9 from ADE Magnetics, with field range ±2 T. The magnetization technique was used to determine the saturation magnetization of the NPs and the magnetic particle volume fraction of the prepared samples, using the following formula:
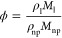
3where ρ_l_ stands for liquid density, ρ_np_ magnetic nanoparticle density, *M*_l_ the specific magnetization of the sample, and *M*_np_ the magnetic nanoparticle specific magnetization.

Room-temperature electron spin resonance (ESR) measurements were performed using an X-band electron magnetic resonance spectrometer (EMX-Plus, Bruker, Rheinstetten, Germany) equipped with a 4119-HS resonant cavity. Samples with a fixed volume (20 μL) at different dilutions were introduced into capillary tubes with an internal diameter of 1 mm. As described in other works,^31^ all spectra were acquired using the following instrumental settings: microwave power, 0.2 mW; modulation frequency, 100 kHz; modulation amplitude, 5 G; magnetic field scan, 6000 G; sweep time, 60 s; and sample temperature, 25 °C.

The MRI measurements were performed at 20 °C with a 1.0T M7 Compact MRI system from Aspect Imaging. Samples were diluted in 14 mL of water from its original batch to obtain concentrations in the range of 0.06 mM to 0.18 mM of [Mn]+[Fe], in order to prevent susceptibility related artifacts on image reconstruction. T1 determination used a inversion recovery pulse sequence with short echo time (TE = 1.9 ms), to reduce T2 influence on the MRI signal intensity. Inversion times varied from 25 to 800 ms with repetition time TR = 4500 ms, flip angle FA = 10°, and voxel dimension of (0.75 × 0.75 × 1) mm^3^. T2 is obtained using a spin echo pulse sequence with high repetition time (TR = 4000 ms), to minimize the effect of longitudinal relaxation T1 on the signal. Same voxel dimension was used. Echo times varied from 7.5 to 25 ms.

### IV.E Thermal Therapies

Near-infrared homemade fiber optic laser system was developed using a commercial diode laser bought from Zhuhai AIKE Photonics Technology, China, that had a SMA-905 fiber connector. The 808 nm laser diode has core diameter of 400 μm and a maximum power of 15 W. The system has a current controller board that tunes the laser power. On the front panel of the system shows a display that shows the voltage and current supplied to the diode, an on/off key, and a button to adjust the current supplied to the laser. The fiber optical cable was bought from Thorlabs, New Jersey, United States and is coupled to the laser through the SMA905 connector. The fiber optic has a length of 2 m with diameter of 200 μm.

The sample holder contained 100 μL of the nanocarrier dispersion with a magnetic nanoparticle concentration of 1 mg/mL. The photothermal properties of the nanocarrier were investigated at 300 mW. The laser optical fiber is positioned on the top of the sample. The temperature was monitored using an infrared thermal camera bought from FLIR, model SC620, Wilsonville, United States. A region of interest (ROI) centered at the laser spot on the sample was used to report the mean temperature during PTT.

The photothermal conversion efficency (PCE) is determined using the equation
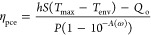
4where *h* is the heat transfer coefficient, *S* is the surface area of the sample, *T*_max_ is the temperature achieved at the stationary regime with laser irradiation, *T*_env_ is the environment temperature, *P* the laser power, and *A*(ω) the absorbance of the sample at the laser wavelength. The parameter *Q*_o_ represents the amount of heat absorbed by the liquid carrier and sample holder that can be determined experimentally. PCE is determined using the Roper’s method.^60^ τ is estimated from the PTT cooling curve, just after turning off the laser.^16^

Magnetic hyperthermia experiments are performed using a MagneTherm system bought from Nanotherics, United Kingdom. It contained a water-cooled induction coil with a diameter of 50 mm (17 turns). The magnetic nanostructures are placed in the center of the sample holder, with a volume of 150 μL. The frequency of the alternating magnetic field is set to 325 kHz. MNH is measured at distinct field amplitudes, at clinical relevant conditions (*Hf* < 5 × 10^9^*A* m^–1^s^–1^). The temperature was monitored with a fiber optic probe bought from LumaSense Technologies, USA. The MNH efficiency is determined using the equation
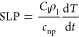
5*C*_l_ and ρ_l_ are, respectively, the heat capacity and the density of the liquid carrier (water), *c*_np_ is the magnetic nanoparticle concentration, and  is the initial heat rate.

### IV.F Cell Culture, Cell Viability, Cellular Uptake, and Immunofluorescence Assays

B16-F10, GL261-eGFP, GL261-mCherry and J774.1 cells were maintained in low-glucose Dulbecco’s modified Eagle’s medium (DMEM, Gibco, USA) supplemented with 10% (v/v) fetal bovine serum (FBS, Gibco, USA), 100 IU/mL penicillin (Gibco, USA), and 100 μg/mL streptomycin (Gibco, USA) and incubated at 37 °C with 5% CO_2_.

We used The CellInsight CX7 LZR Pro (ThermoFisher) to acquire immunofluorescence assays in the dark edges of 96 well plates (Greiner) images. The cells were exposed to formulations previously incorporated with IR780 at a final concentration of 0.125 mg/mL and maintained in a cell cultivation oven at 37 °C at 5% of CO_2_ for 1 h. After exposure, the medium containing the formulation was removed and three washes with 100 μL of PBS were performed, followed by 10 μg/mL of WGA (Wheat Germ Agglutinin – Alexa Fluor 488) for labeling cell membranes and 1 μg/mL of Hoechst 33342 to label cell nuclei for 10 min at 37 °C. The red fluorescence was used for the identification of GL261-mCherry fragments, purple for formulations labeled with IR780, cell membranes identification was green (also for GL261-eGFP) and blue for cell nuclei.

The cells (GL261, B16F10 and J774A1) were seeded on 12 well-plates at a density of 1 × 10^5^ cells per well and incubated at 37 °C and 5% CO_2_ overnight. Fluorescent formulations (labeled with Liss Rhod PE, Excitation 560 nm, emission 583 nm) diluted with the media to final concentration of 0.125 mg/mL were applied in each well for 4 h. Then, cells were washed with PBS, to remove residual formulations, detached using trypsin and centrifuged with PBS at 1.5 × *g* for 5 min. Intracellular uptake was monitored using the BD FACSCanto II flow cytometer, evaluating 10,000 events for each analysis. The B16-F10, J774.1, and GL261-mCherry cell lines were initially distributed in 96-well plates at 1 × 10^4^ cells/well and allowed to attach overnight and then subjected to different incubation temperatures. Then, the MTT assay was used to measure cell viability. Cell viability of temperature-treated cells was normalized to the viability of the control temperature group (°C). Cell viability was also evaluated by flow cytometry. In this case, 1 × 10^5^ cells were seeded per well and incubated at 37 °C and 5% CO_2_ overnight. Dilutions of the formulations, NP (magnetic nanoparticles) and MLs (magnetoliposomes), were carried out and applied to cells for 24h incubation at 37 °C and 5% CO_2_. Over again, cells were washed with PBS, to remove residual formulations, detached using trypsin and centrifuged with PBS at 1.5 × g during 5 min. Cell were stained with vital dye propidium iodide (PI), that is cell membrane impermeable and stains only dead cells. Differentiation among cell populations were performed using a BD FACS Canto II flow cytometer (Becton Dickinson & Company, USA). All the data obtained by flow cytometry was analyzed using the FCS Express software program (De Novo Software, USA).

### IV.G *Ex Vivo* Experiments

Small pork loin pieces were subjected to a multi slice T1W spin echo pulse sequence with TR = 600 ms, TE = 7 ms, and voxel dimension of (0.5 × 0.5 × 0.5) mm^3^ before and after injection of 120 μL of high and low concentration ML5.0_IR780 aliquots.

Tomographic fluorescence imaging was perfomed after the injection of the near-infrared ML into the biomaterial, pork loin. The FMT Imaging System was bought from PerkinElmer (Waltham,MA), and operates at four channels, namely: 635, 680, 750, and 790 nm. The fluorescence studies of this work were performed using a 790 nm excitation channel with a maximum laser output power of 80 mW. 3D reconstruction and merge of both MRI and FMT scans were performed with the 3D Viewer plugin from ImageJ software. The high concentration sample was subjected to a PTT trial.

### IV.H *In Vivo* Experiments

Female C57Bl/6 mice, 6 to 8 weeks old and with an average body weight between 25 and 35 g, were used for the *in vivo* studies. The animal maintenance conditions and experimental procedures for the animal study, as well as anesthesia (isoflurane) and euthanasia protocols, were reviewed and approved by the Ethics Committee for the Use of Animals (CEUA) at the Federal University of Goias under protocol 109/22. Cells of the B16F10 murine melanoma lineage were obtained from the Rio de Janeiro Cell Bank (BCRJ, Rio de Janeiro, Brazil). Solid tumors were induced in the dorsal region of the animals by inoculating, through subcutaneous injection, 50 μL of solution with 1 × 10^6^ viable cells. Tumor volume measurements were performed with a digital caliper daily. Tumor volume was calculated as follows:
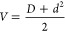
6where *D* is the long axis and *d* is the short axis of the solid tumor in mm. 50 μL of ML5.0_B16_IR780 was injected into the lateral tail vein in the intravenous study (I.V.). For the intratumoral study (I.T.), 100 μL was administered, while for the intraperitoneal study (I.P.) 200 μL was injected into the peritoneal region. Animals were subjected to a series of coronal T1W spin echo pulse sequence with TR = 600 ms, TE = 10 ms, and voxel dimension of (1.0 × 1.0 × 1.0) mm^3^ throughout the study. After euthanasia, the organs were collected and subjected to a GRE pulse sequence with TR = 80 ms, TE = 3.2 ms, and voxel dimension of (0.5 × 0.5 × 2.0) mm^3^. The photothermal (PTT) experiments used a diode laser with 808 nm wavelength, model Laser iZi 808, bought from LASERline (Sao Paulo, Brazil). The surface temperature was monitored using the infrared thermal camera. For PTT, FMT and MRI, the animals were kept anesthetized by isoflurane, 4–5% for induction and 1–2% for maintenance.

## Supporting Information Available

MnFe_2_O_4_ synthesis description, EDS compositional information on a ML sample, TEM image of the hybrid vesicles, DLS data of all systems, spin echo image of the ML with different concentration, cell viability, MNH parameters used in SLP calculation, photothermal stability experiment, 3D reconstruction of FMT *ex vivo*, MNH *ex vivo* study of temperature variation, IT *in vivo* experiment data, GRE MRI images of organs in I.V. *in vivo* experiments, I.P. *in vivo* T1W MRI images, and GRE MRI images of the organs in the I.P. *in vivo* experiment (PDF)
3D images at different positions (MP4)
