# Supplementary material for: Near Infrared Biomimetic Hybrid Magnetic Nanocarrier for MRI-Guided Thermal Therapy
Source: ACS Appl Mater Interfaces. 2024 Jul 8;17(9):13094–110. doi: 10.1021/acsami.4c03434 (PMC11891835; doi:10.1021/acsami.4c03434)
Supplement: Supplementary file 1 — am4c03434_si_001.pdf [file am4c03434_si_001.pdf]

Supporting Information: Near infrared biomimetic hybrid magnetic nanocarrier for MRI-guided thermal therapy

João Victor Ribeiro Rocha,[1] Rafael Freire Krause,[1] Carlos Eduardo Ribeiro,[1] Nathália Corrêa de Almeida Oliveira,[2] Lucas Ribeiro de Sousa,[1] Juracy Leandro Santos Junior,[1] Samuel de Melo Castro,[1] Marize Campos Valadares,[3] Mauro Cunha Xavier Pinto,[4] Marcilia Viana Pavam,[2,5] Eliana Martins Lima,[2,5] Sebastião Antônio Mendanha,[1,2,5] and Andris Figueiroa Bakuzis[1,5,*]

[1]*Institute of Physics, Federal University of Goiás, Goiânia, GO 74690-900, Brazil*
[2]*FarmaTec – Laboratory of Pharmaceutical Technology, Federal University of Goiás, Goiânia, GO 74690-631, Brazil*
[3]*ToxIn – Laboratory of Education and Research in In Vitro Toxicology, Federal University of Goiás, Goiânia, GO 74690-631, Brazil*
[4]*Department of Pharmacology, Institute of Biological Sciences, Federal University of Goiás, Goiânia, GO 74690-900, Brazil*
[5]*CNanoMed – Nanomedicine Integrated Research Center, Federal University of Goiás, Goiânia, GO 74690-631, Brazil*

(Dated: June 11, 2024)

I. MAGNETIC NANOPARTICLE SYNTHESIS

The formation of manganese ferrite follows the following reaction equation:

$$1Mn^{2+} + +Fe^{3+} + +8OH^{-} MnFe_2O_4 + 4H_2O \tag{1}$$

The masses of the reagents required for this synthesis were determined based on reaction stoichiometry. A solution of 90 ml methylamine (CH_3NH_2) from Sigma-Aldrich, diluted in 400 ml distilled water, served as the medium base. This basic solution was heated to its boiling point while being stirred. The Mn2+ and Fe3+ ions were provided by 0.5 mol/L solutions of $MnCl_2.4H_2O$ and 1 mol/L of $FeCl_3.6H_2O$, respectively.

For the Mn2+ solution, 98.95 g of $MnCl_2.4H_2O$ was diluted in 50 ml of HCl and further diluted in 950 ml of water. The Fe3+ solution involved using 270.30 g of $FeCl_3.6H_2O$, followed by dilution with 50 ml of HCl and further dilution in 950 ml of water. Equal volumes (50 ml) of the Mn2+ and Fe3+ solutions were mixed and poured over the heated CH_3NH_2 solution, leading to the formation of a dark-colored precipitate. After stirring for thirty minutes, the particles were magnetically separated and washed three times with distilled water.

The passivation process involved separating the particles magnetically, removing water, and washing with a 0.5 M solution of nitric acid (HNO_3). Subsequently, a 0.5 mol/L solution of iron nitrate ($Fe(NO_3)_3$) was applied, stirring and heating for 30 minutes before cooling. Magnetic separation, removal of supernatant, washing with acetone three times, and allowing acetone to evaporate followed this step.

The final passivated nanoparticles were coated with citrate ions to produce a stable colloid resistant to agglomeration and precipitation. Sodium citrate ($Na_3C_6H_5O_72H_2O$) was added to the dispersion, with a ratio of 1 mol for every 10 mol of iron (Fe). The dispersion was stirred and heated at 80 °C for 10 minutes, cooled at room temperature, and the coated nanoparticles were magnetically separated. After discarding the supernatant, the coated nanoparticles were washed with acetone three times. Once acetone evaporated, the citrate-coated $MnFe_2O_4$ nanoparticles were resuspended in distilled water, completing the magnetic fluid preparation.

* bakuzis@ufg.br

II. CHARACTERIZATION

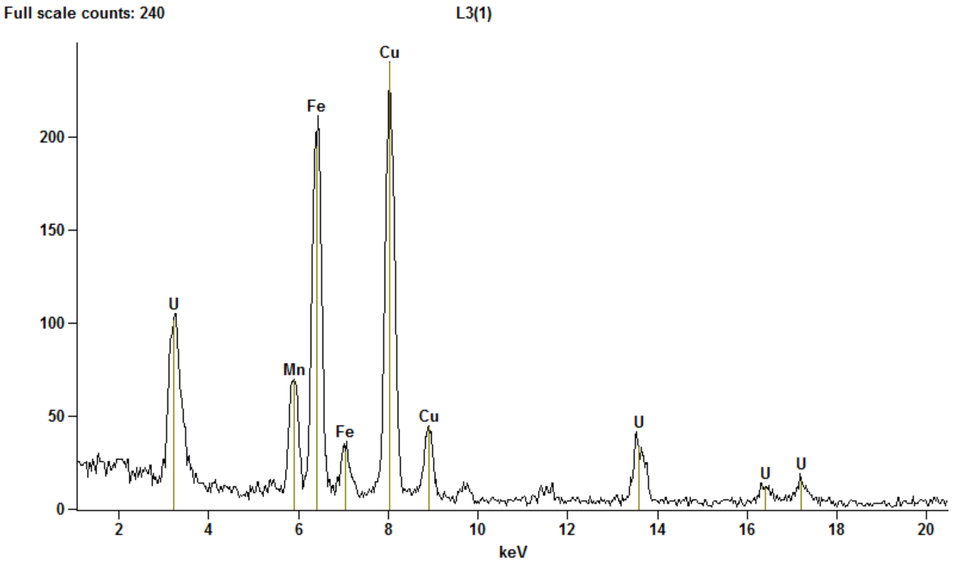

Figure S1.: Compositional information of a ML sample, obtained by Energy-dispersive X-ray spectroscopy (EDS) to illustrate the chemical/elemental characterization of the nanocarriers used in this work. The position of the peaks in the spectrum identifies the element, whereas the intensity of the signal corresponds to the concentration of each element. Fe and Mn peaks are related to the magnetic NP composition while U and Cu come from the stain (uranyl acetate) and from the electron microscopy grid, respectively.

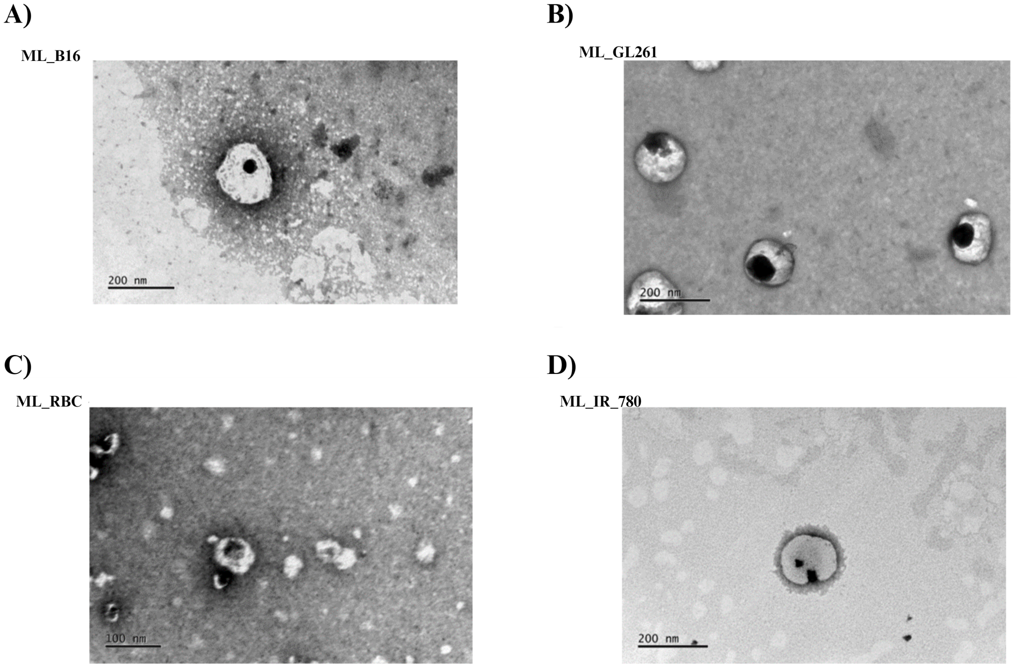

Figure S2.: Illustrative TEM images of the magnetoliposomes and hybrid vesicles developed in this work. Panels (A), (B), (C) and (D) shows images of ML5.0-B16, ML5.0-GL261, ML5.0-RBC and ML5.0-IR biomimetic liposomes, respectively. The high contrast (black) regions indicate the presence of magnetic NPs inside the vesicles. These images were acquired using a JEOL JEM 1011 electron microscope, able to work at 80 kV.

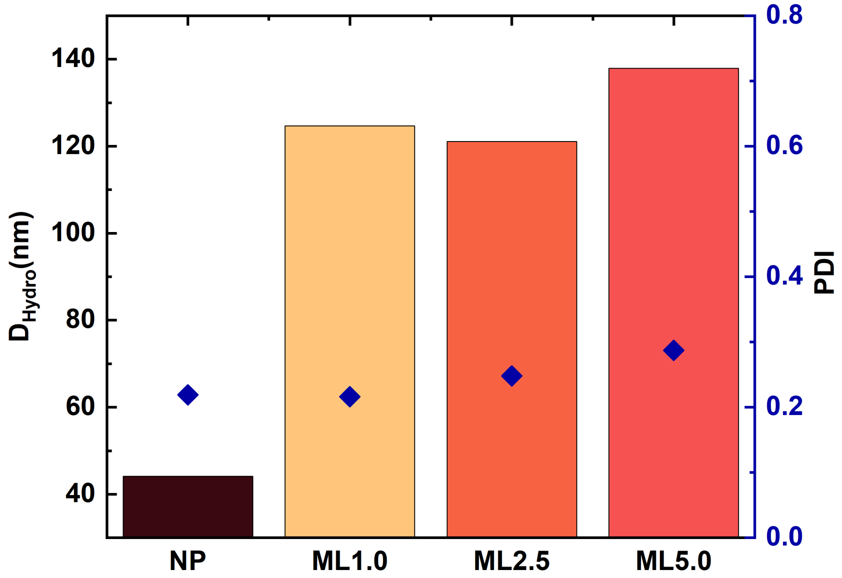

Figure S3.: Dynamic Light Scattering (DLS) hydrodynamic diameter as well as the polydispersity index (PDI) of NP and MLs prepared by freeze and thaw cycles followed by extrusion using polycarbonate filters. Diameters of 120-140 nm and PDI $<$ 0.3 indicate the success and reproducibility of the this sample preparation methodology. The diameters values were used to evaluate the NPs organization/encapsulation inside each liposome.

III. MAGNETIC RESONANCE IMAGING

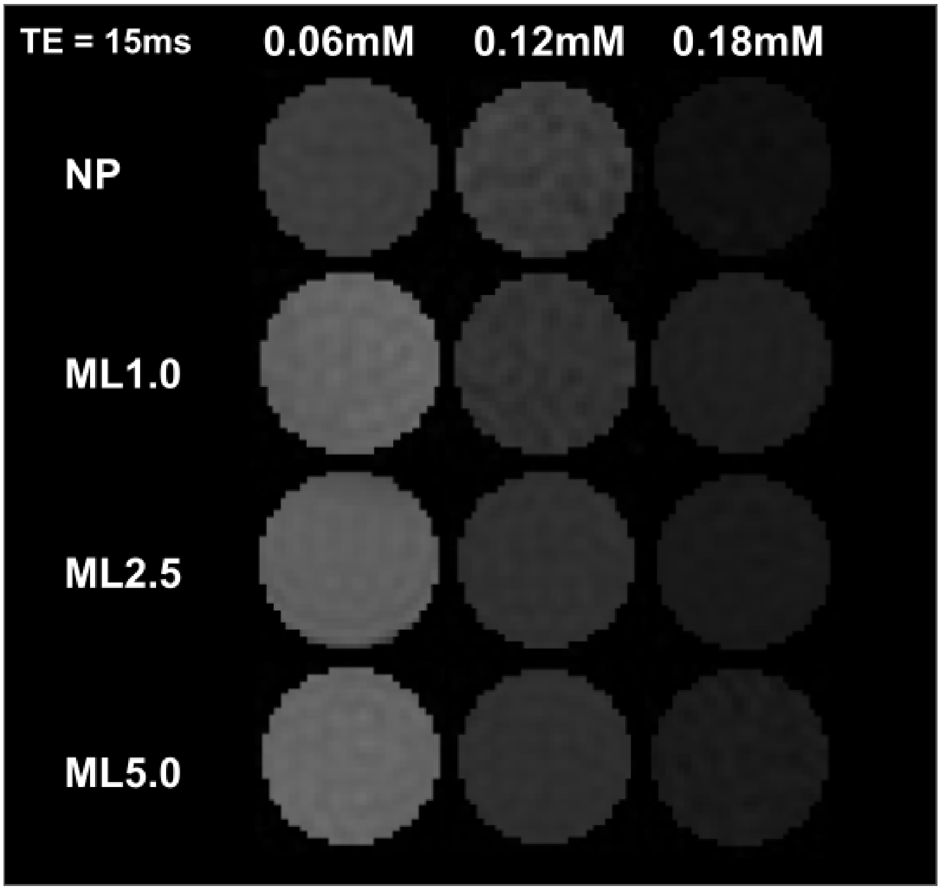

Figure S4.:Spin Echo images with fixed echo time TE = 15ms. In this case, we are comparing the performance of synthetic ML's and the NP at different particle concentration.

IV. IN VITRO EXPERIMENTS

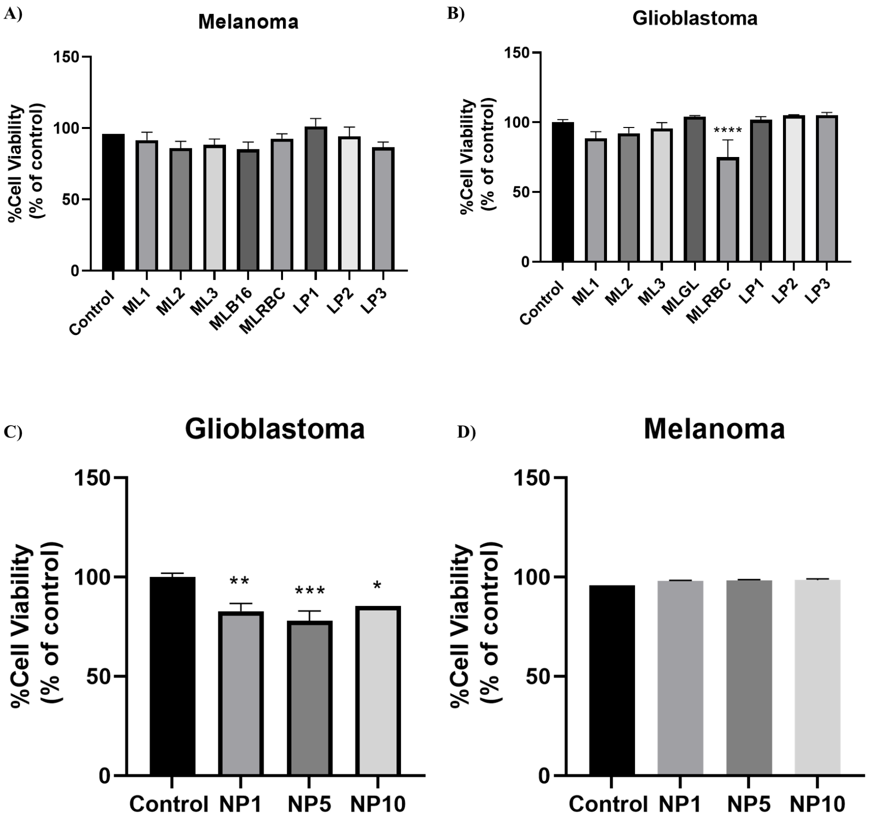

Figure S5.: Determination of cell viability by PI staining and flow cytometry demonstrating the low cytotoxicity of magnetic NPs as well as of all magnetoliposomes used in this work. Control liposomes (LP1-LP3) containing no NPs were also included to demonstrate the safety of liposomal formulations. Melanoma (Panels A and D) and Glioblastoma (Panels B and C) cells were treated with magnetic NPs or liposomes for 24 h before viability evaluation. The living cells population was determined as the percentage of PI non-stained cells. Cell viability was expressed as percentage of controls. Data are expressed as the means ± SD (n = 3). $p < 0.05$; **$p < 0.005$; **$p < 0.001$.. Magnetoliposomes were selected according their NP concentration (mg/mL): ML1 = 0.250; ML2 = 0.125; ML3 = 0.062 and MLB16 = MLRBC = MLGL = 0.250. Control liposomes were tested according their lipid concentration (mM): LP1 = 2.0; LP2 = 1.0 and LP3 = 0.5. Finally, NP1 = 1 mg/mL; NP5 = 5 mg/mL and NP10 = 10 mg/mL.

V. MAGNETIC HYPERTHERMIA

The model behind the fitting of SLP and frequency is based on the linear response theory. In this case, when low frequency fields are used, the SLP can be determined by

$$SLP_{theo} = \frac{\mu_0 \pi \chi_0 H_0^2}{\rho} \frac{(2\pi f^2 \tau)}{(1 + (2\pi f \tau)^2)} \tag{2}$$

where ρ, χ_0, τ are, respectively, the magnetic material density, equilibrium susceptibility and effective magnetic relaxation time. Moreover, the SLP could be fitted following the equation below:

$$SLP_{fit} = A \frac{(f^2 \tau)}{(1 + (2\pi f \tau)^2)} \tag{3}$$

The parameters A and τ for the given curves can be found in Table S1.

	**Samples**					
	**NP**	**ML1.0**	**ML2.5**	**ML5.0**	**ML7.5**	**ML10.0**
**τ ($\times$ $10^{-}7$ s)**	**4.3 ± 1.5**	**4.6 ± 0.4**	**6.4 ± 2.0**	**4.4 ± 0.7**	**7.6 ± 1.7**	**6.8 ± 0.5**
**A ($\times$ 10^{-4} J/g)**	**8.7 ± 1.7**	**27.1 ± 1.1**	**13.8 ± 1.9**	**12.5 ± 1.1**	**14.2 ± 1.5**	**14.5 ± 1.4**

Table S1.: τ and A values obtained via fitting according to Equation 3.

VI. PHOTOTHERMAL STABILITY

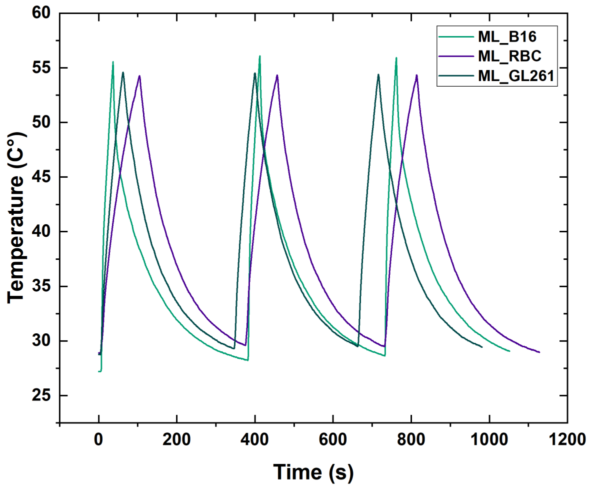

Figure S6.: Photothermal stability experiments for biomimetic-IR780 labeled magnetoliposomes containing 2.5mg/mL of magnetic nanoparticles. Three cycles of laser irradiation were used to illustrate the ability of biomimetic vesicles in repeatedly generating heat through photostimulation. Although IR780 molecules could also contribute to the overall heat generation, the main contribution to the nanocarriers photothermal response is related to the magnetic NPs. Thus, no photobleaching is expect and the irradiation cycles might be repeated indefinitely. Parameters: laser power, 500 mW; exposure time, 90 sec; maximum temperature 55 °C.

VII. MRI AND FLUORESCENCE MOLECULAR TOMOGRAPHY

As said, a pork loin was injected with a ML5.0_ IR780 sample. The NP concentration was 5 mg/mL and the IR780 concentration was 150 μ g/mL. MRI T1-Weighted images were performed as well as fluorescence molecular tomography essays. The 3D reconstruction is available in this link.

VIII. *EX VIVO* MNH STUDY

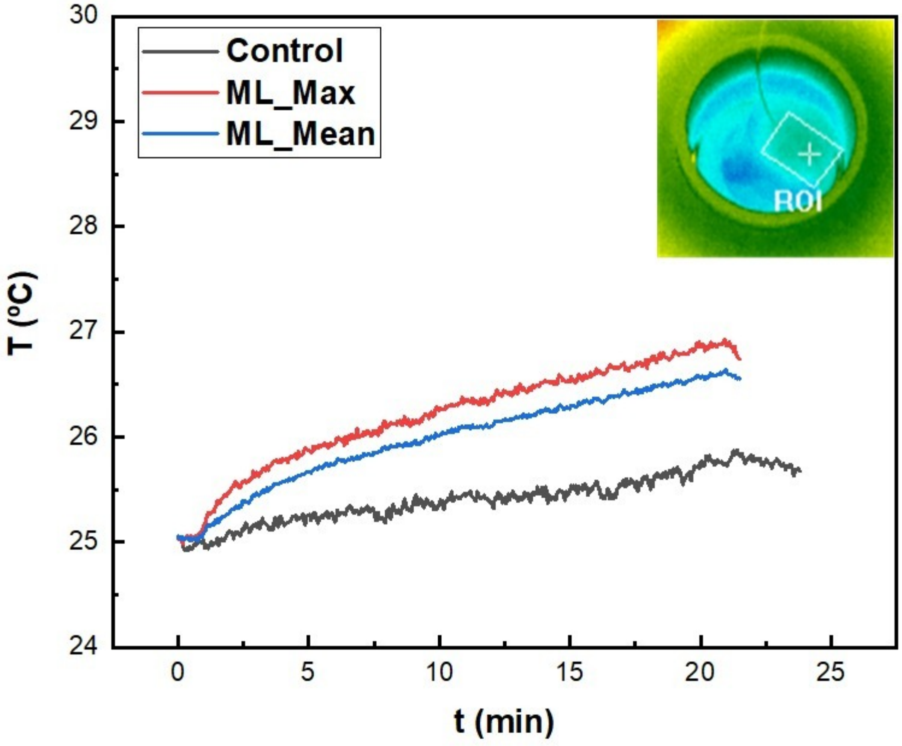

Figure S7.: MNH study in the pork loin showing no significant temperature variation at the surface. 500μL injection of ML5.0-IR780 was injected 5 mm below the surface of pork loin. The magnetic field frequency was 323 kHz and the field amplitude 86 Oe

IX. IN VIVO STUDIES

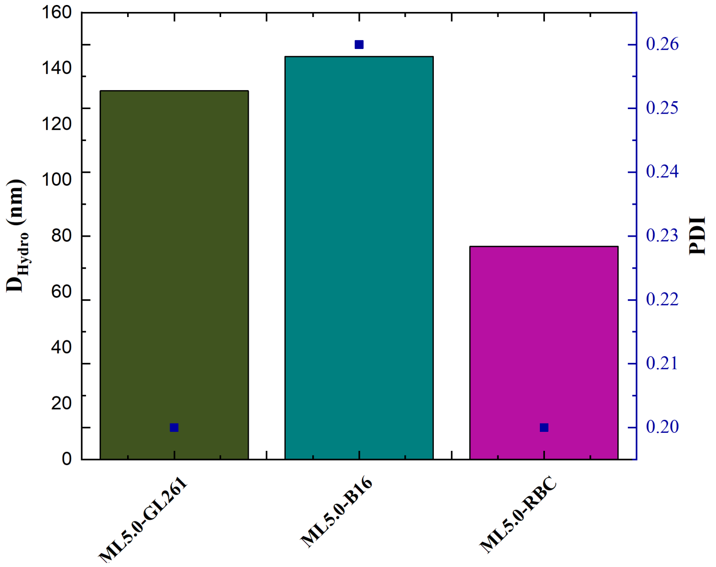

Figure S8.: Hydrodynamic diameter and polydispersity index of biomimetic magnetolipossome used in the *in vivo* experiments. All the data was obtained by Dynamic Light Scattering (DLS). In this case MLs were prepared using freeze and thaw cycles followed by a sonication protocol. Notably, diameters of 80-140 nm and PDI < 0.3 indicate the success and reproducibility of the this methodology. We opted by use the sonication procedure to prepare the magnetoliposomes containing high magnetic NPs concentration because this methodology better preserves the lipid and protein content of the biomimetic nanocarriers in comparison to the extrusion procedure.

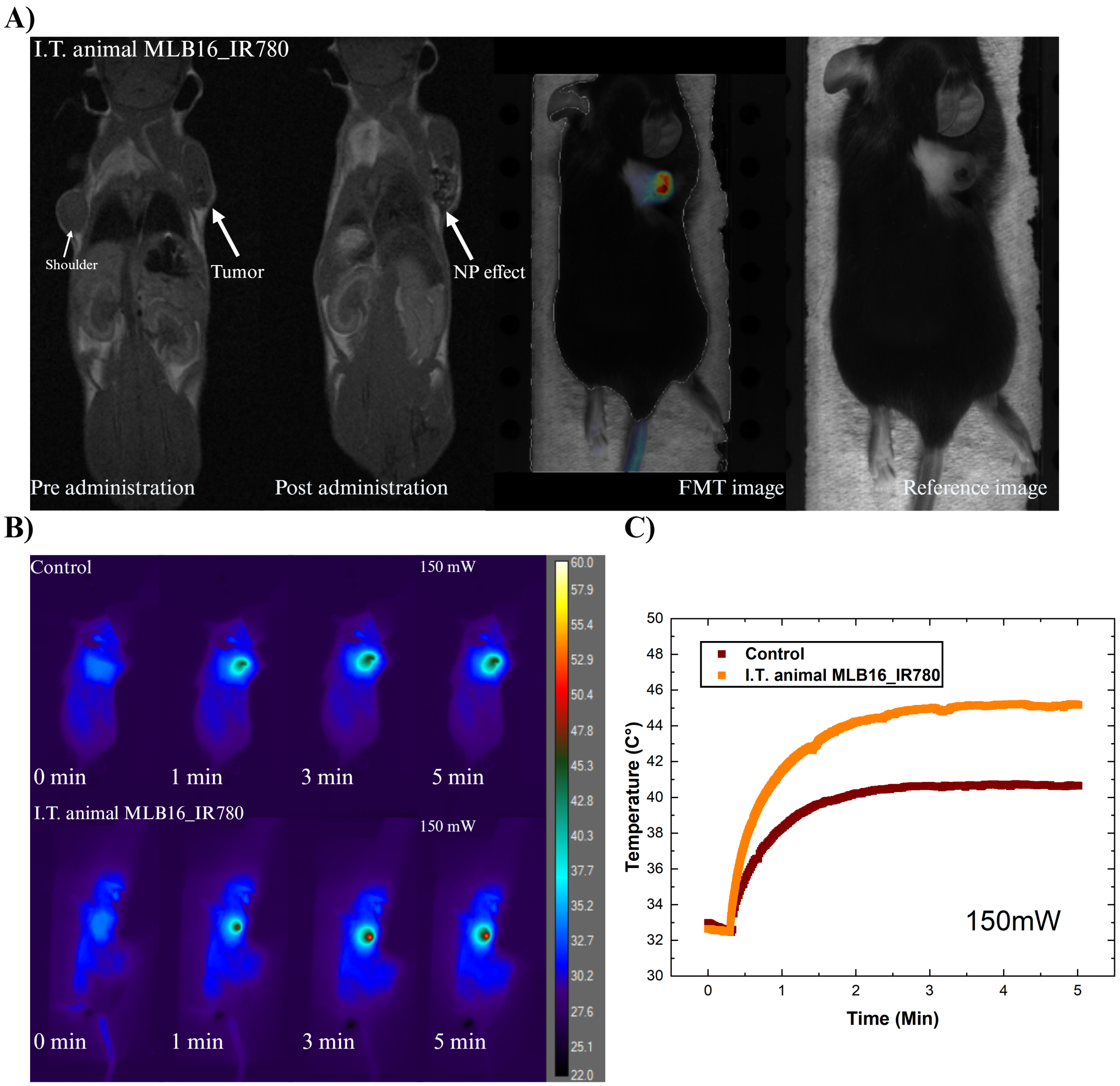

Figure S9.: The intratumoral administration of 100 μL of the biomimetic liposome ML1.0_GL261_IR780 with a magnetic particle concentration of 2.5 mg/mL was used as a proof of concept to evaluate the photothermal therapy properties of the nanocarrier. Panel (A) shows the T1w MRI image pre- and post-administration of the magnetic nanoparticles. One can see after administration the loss of contrast in the tumor region due the presence of NPs (see arrows). FMT image also confirm the presence of the near-infrared nanocarrier due to the IR780 dye incorporated in the hybridized vesicles. Panel (B) shows images obtained by the thermal camera during PTT treatment with and without (control) NPs injection. It is possible to see that the maximum temperature in the tumor was higher after hybrid vesicle injection. Panel (C) shows the tumor temperature profile obtained during PTT treatment for both situations, pre and post intratumoral administration.

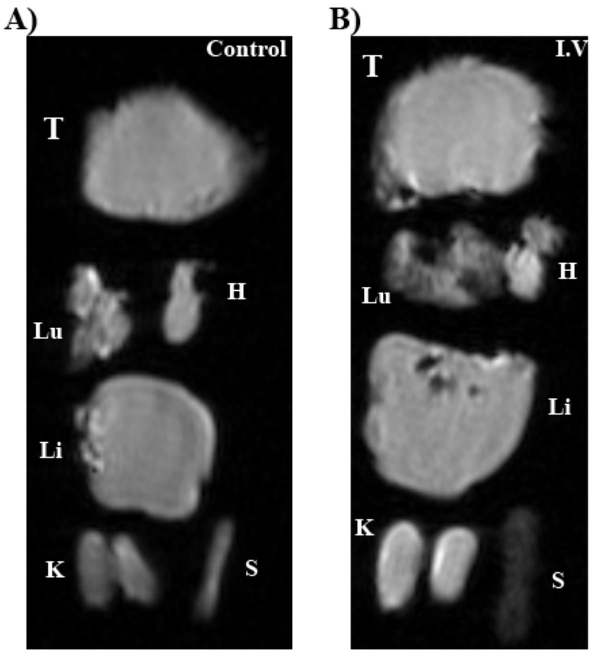

Figure S10.: (A) GRE MRI images of the organs and tumor of the animal that did not received the hybrid liposome injection (control). (B) MRI images of the organs and tumor of the animal that received 50 μL of ML1.0_GL261_IR780 by intravenous (I.V.) injection with a magnetic particle concentration of 2.5 mg/mL. T stands for tumor, Lu for lungs, H for heart, Li for liver, K for kidneys and S for spleen.

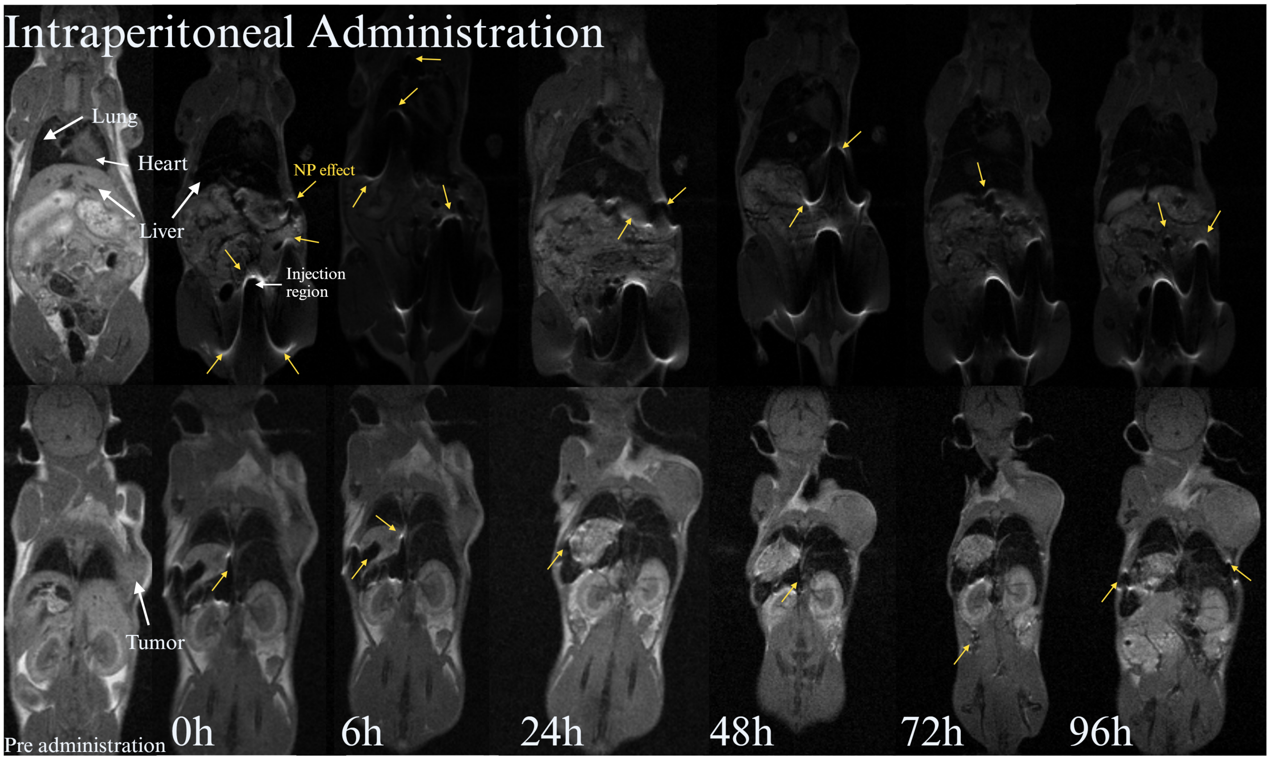

Figure S11.:The panel show two slices of MRI images in animals that received intraperitoneal administration of 200 μL of ML5.0-B16-IR780 nanoparticles with a magnetic particle concentration of 2.5 mg/mL. The upper images show the lower part of the animal's body, where it is possible to see a higher effect of the NPs accumulation on several organs. The lower images show the upper part of the animal's body, where it is possible to see the tumor and NPs accumulation. Arrows were used to point at the visible effects of loss of contrast due the presence of NPs.

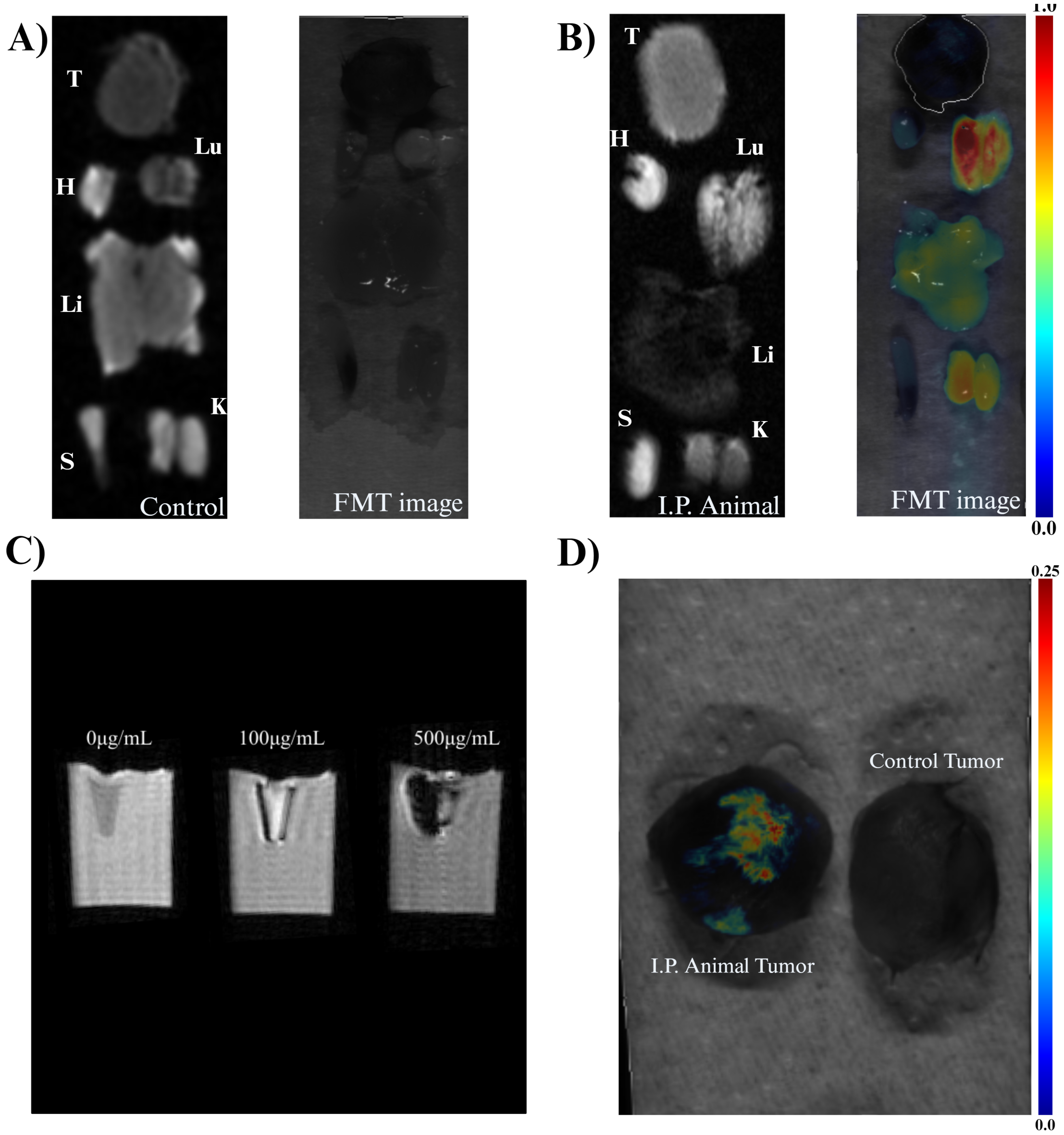

Figure S12.: (A) Gradient Recall Echo (GRE) MRI and Fluorescence Molecular Tomography (FMT) images of the tumor and organs extracted from the animal that did not received NPs intraperitoneal (I.P.) injection. (B) GRE-MRI and FMT images of the tumor and organs extracted from the animal that received intraperitoneal injection of ML5.0-B16-IR780 nanoparticles (200 μL). Higher NP concentrations promotes loss of signal intensity and spatial resolution in GRE-MRI images, as observed in the liver (Li), while small concentrations results on a brighter signal, as seen in the heart (H) and the tumor (T). Additionally, the fluorescent signal of IR780 incorporated into ML5.0-B16 membranes confirm the nanocarrier accumulation on the aforementioned organs and tumor. Panel (C) shows GRE-MRI images magnetic particle concentration effect considering an *in vitro* study. As observed for *in vivo* images, lower NP concentrations preserve spatial resolution and improves signal intensity, on the other hand, higher NP concentrations result in both loss of signal intensity and spatial resolution. (D) FMT image for a normalized signal intensity scale showing the fluorescent emission of the tumor extracted from the animal that received I.P. biomimetic liposome injection, demonstrating the nanocarrier accumulation. The tumor of the control animal (no injection) was included for signal comparison.
